# Human Endogenous Retrovirus Type W Envelope from Multiple Sclerosis Demyelinating Lesions Shows Unique Solubility and Antigenic Characteristics

**DOI:** 10.1007/s12250-021-00372-0

**Published:** 2021-03-26

**Authors:** Benjamin Charvet, Justine Pierquin, Joanna Brunel, Rianne Gorter, Christophe Quétard, Branka Horvat, Sandra Amor, Jacques Portoukalian, Hervé Perron

**Affiliations:** 1 GeNeuro Innovation, Lyon, 69008 France; 2 CIRI, International Center for Infectiology Research, INSERM U1111, CNRS UMR5308, University of Lyon, ENS Lyon, France; 3 Université Claude Bernard Lyon 1, Lyon, 69000 France; 4 Department of Pathology, Amsterdam UMC, Location VUMC, 1007 MB Amsterdam, The Netherlands; 5 ProteinSimple, Abingdon, OX14 3NB UK; 6 Centre for Neuroscience and Trauma, Blizard Institute, Barts and London School of Medicine and Dentistry, Queen Mary University of London, London, E1 2AT UK

**Keywords:** Multiple sclerosis (MS), Brain, Antigen, HERV-W, Envelope, Endogenous retrovirus, MSRV-ENV, Syncytin, Demyelination, Lipids, Glycolipids, Sulfatides, Oligomer, Hexamer

## Abstract

In multiple sclerosis (MS), human endogenous retrovirus W family (HERV-W) envelope protein, pHERV-W ENV, limits remyelination and induces microglia-mediated neurodegeneration. To better understand its role, we examined the soluble pHERV-W antigen from MS brain lesions detected by specific antibodies. Physico-chemical and antigenic characteristics confirmed differences between pHERV-W ENV and syncytin-1. pHERV-W ENV monomers and trimers remained associated with membranes, while hexamers self-assembled from monomers into a soluble macrostructure involving sulfatides in MS brain. Extracellular hexamers are stabilized by internal hydrophobic bonds and external hydrophilic moieties. HERV-W studies in MS also suggest that this diffusible antigen may correspond to a previously described high-molecular-weight neurotoxic factor secreted by MS B-cells and thus represents a major agonist in MS pathogenesis. Adapted methods are now needed to identify encoding HERV provirus(es) in affected cells DNA. The properties and origin of MS brain pHERV-W ENV soluble antigen will allow a better understanding of the role of HERVs in MS pathogenesis. The present results anyhow pave the way to an accurate detection of the different forms of pHERV-W ENV antigen with appropriate conditions that remained unseen until now.

**Supplementary Information:**

The online version contains supplementary material available at 10.1007/s12250-021-00372-0.

## Introduction

Human endogenous retroviruses (HERVs) represent about 8% of the human genome (Feschotte and Gilbert 2012; Grandi and Tramontano 2018b) and, along with other multicopy transposable elements represent nearly one-half of human DNA (Pace and Feschotte 2007; Hancks and Kazazian 2016), compared to the small proportion (< 3%) of sequences encoding physiological genes (Medina and Perron 2017). These remnants of “mobile genetic elements” mostly comprise defective or non-coding copies. They nonetheless comprise coding or regulatory sequences that have been co-opted for physiological functions (Jacques *et al.* 2013) or retained a coding and pathogenic potential (Kazazian and Moran 2017; Bourque *et al.* 2018; Kremer *et al.* 2019a; Payer and Burns 2019). HERVs entered the genome of species via infections of germline cells by exogenous retroviruses and insertion of their DNA copies (proviruses) in chromosomes. Copies still retaining coding and functional properties may therefore express many characteristics of retroviruses, which may occur after modifications of their epigenetic control and activation of their transcription by environmental factors (Perron and Lang 2010; Charvet *et al.* 2018). This may explain why HERVs are increasingly incriminated in complex and multifactorial diseases for which no simple infectious nor genetic factor can recapitulate the pathogenesis and the epidemiology (Perron and Lang 2010; Kazazian and Moran 2017; Kury *et al.* 2018).

The HERV-W family was unveiled and characterized within human DNA following the identification of novel retroviral sequences from virus-like particles isolated in cell cultures from people with multiple sclerosis (MS), a progressively disabling neurological disease affecting millions of individuals worldwide (Perron *et al.* 1997b). The prototype sequence from DNA-free RNA of purified retroviral particles (then named MSRV) was not recorded in nucleic acid databases (Perron *et al.* 1997b; Komurian-Pradel *et al.* 1999). Although the whole human genome was reported to be sequenced, this missed regions such as centromeres, and the HERV-W chromosomal copy identical to MSRV sequences was not seen. Only a defective copy in chromosome 7 (ERVWE1) was found to encode an HERV-W envelope protein, also named syncytin-1 because physiologically “domesticated” to play a critical role in the fusion of syncytio-trophoblast cells of the placenta (Blond *et al.* 2000; Dupressoir *et al.* 2012) and of osteoclasts during bone formation (Soe *et al.* 2011). Another copy in chromosome-X (ERVWE2) showed sequences identical to MSRV envelope gene but with an in-frame stop codon, no intact gag-pol genes and a non-functional proviral promoter (Roebke *et al.* 2010; do Olival *et al.* 2013). Nonetheless, MSRV sequences and the corresponding proteins have been detected and associated with MS by different groups in independent studies as reviewed (Morandi *et al.* 2017; Kury *et al.* 2018). Its envelope protein, first called “MSRV-ENV” and now named pathogenic envelope “pHERV-W ENV”, was shown to reproduce the hallmarks of MS pathogenesis in studies with *in vitro* and *in vivo* models of MS, in parallel with brain immunohistology observations (Perron *et al.* 2012; Perron *et al.* 2013; Kremer *et al.* 2013; Kremer *et al.* 2019a; van Horssen *et al.* 2016).

In DNA studies of human populations, the existence of non-ubiquitous and/or unfixed HERV proviruses has recently been evidenced for the HERV-K (Wildschutte *et al.* 2016), HERV-W and HERV-H families (Thomas *et al.* 2018). This indicates that certain HERV copies are not present in all individuals, thus creating heterogeneity in human genome(s). The presence of such copies may thus be associated with disease susceptibility although their identification requires appropriate technical approaches since recent sequencing technologies do not allow an exhaustive identification of homologous and multicopy HERV sequences (Thomas *et al.* 2018; Marks *et al.* 2019). Present genome sequencing methods neither address somatic DNA rearrangements within a limited number of affected cells, from brain lesions or from blood, which produce HERV proteins in disease (Anderson *et al.* 2018).

Similarly, pHERV-W ENV antigen has not been thoroughly characterized and, apart from immunohistochemistry showing detection in active lesions of all MS brains analyzed to date (van Horssen *et al.* 2016; Kremer *et al.* 2019a), it remains difficult to detect with technical conditions suitable for routine and large-scale epidemiological analyses of body fluids. More generally, no diagnostic test has been developed for any (human) retroviral envelope protein to date. This has led to alternative diagnostic detection of, e.g., HIV-1 gag-encoded antigens (Schupbach *et al.* 2000).

Given the pathogenic link of pHERV-W ENV protein with MS, the present study was performed to assess the biochemical and physical properties of pHERV-W ENV protein compared to HERV-W envelope protein from chromosome 7, syncytin-1, and the envelope protein from another endogenous family, HERV-K. The present findings revealed unexpected properties that explain why studies using classical methods, e.g., ELISA immunoassays or mass-spectrometry, are inadequate tools for a standardized, straightforward and routine detection of HERV-W antigens in body fluids. Moreover, amino-acid sequences encoded by the original cDNA clones obtained from MS retroviral particles predicted observed antigenic and physico-chemical characteristics of pHERV-W antigen from MS brain lesions.

## Materials and Methods

### Sequences Alignment

HERV-W *ENV* (accession: AAK18189.1) and *ERVWE1* (accession: AAF28334.1) amino acids sequences were aligned using EMBL-EBI_MUSCLE website (https://www.ebi.ac.uk/Tools/msa/muscle/).

### Oligomerization Tests

Purified recombinant full-length HERV-W ENV protein was produced in *E. coli* and solubilized in (20 mmol/L Tris–HCl pH 7.5, 150 mmol/L NaCl, 1.5% SDS, 10 mmol/L DTT buffer (PX Therapeutics).

Recombinant HERV-W ENV protein was incubated 24 h at 37 °C in DMEM/F12 1× (Gibco, 31,331–028) completed with a combination of several reagents: 1.5% SDS (Sigma, 74,255-250G); 10% fetal bovine serum (FBS) (ATCC, 30–2022); 10% BSA (Sigma; A7906) and 1% fos-cholin 16 (Anatrace, F316S-1GM). Glycolipids were also used in oligomerization tests such as sulfatides from bovine brain (Sigma, S1006-5MG), cholesterol (Sigma, C8503-100G), sphingomyelin from bovine spinal cord (Millipore, 567,706-100MG) and galactocerebrosides (galactosylceramides) from bovine brain (Sigma, C4905-10MG). Because of their poor solubility, sulfatides were diluted in CHCl_3_/MetOH 2:1 (v/v), galactosylceramides in MetOH, and cholesterol or sphingomyelin in CHCl_3_.

### HEK293T Transfected Cells

HEK293T cells were cultured at 37 °C and 5% CO_2_ in DMEM/F-12 (Gibco, 31331-028) supplemented with 10% of heat-inactivated (30 min at 56 °C) bovine FBS (ATCC, 30-2020) and 10 µL Penicillin–Streptomycin/mL (Sigma, P4333). 250,000 HEK cells were transfected with 3 µg of plasmid using Lipofectamine 2000 transfection reagent (Invitrogen, 11668-019). The following constructions under the control of CMV promoter (GeNeuro, Switzerland) were transfected: pMAX-*GFP*, encoding GFP and used as control, pCMV-HERV-W *ENV* encompassing the complete ORF of pHERV-W *ENV* (cDNA clone from MS cell culture virion RNA encoding 542 amino acids GenBank no. AF331500.1), pCMV-*ERVWE1* (cDNA from full-length placenta RNA encoding syncytin-1, 538 amino acids GenBank no. AF208161.1), pCMV-HERV-K *ENV* encompassing the complete ORF of *ENV* from HERV-K113 clone (Beimforde *et al.* 2008; Hanke *et al.* 2009); pCMV-HERV-K *ENV-SU-HP* encompassing the complete ORF of *ENV* -SU from HERV-K113 clone followed by the additional sequence of HP, as described in Supplementary Figure S1. Transfected cells were harvested 24–48 h post transfection.

The HP polypeptide was synthesized according to the amino acid sequence (Supplementary Figure S1), by Smart Bioscience, Saint Egrève, France.

### Brain Samples

Human brain tissue was obtained at autopsy from 13 MS cases and 4 age-matched cases with no neurological disorders. The rapid autopsy regimen of the Netherlands Brain Bank in Amsterdam (coordinator Dr. I. Huitinga) was used to acquire the samples, with the approval of the Medical Ethical Committee of the Amsterdam UMC. All participants or next of kin had given informed consent for autopsy and use of their tissues for research purposes. Tissue samples from MS cases were selected from regions of interest after *ex vivo* MRI (Bo *et al.* 2004). The cases and lesions were selected from a large cohort of MS cases based on the size and lesion type for quantitative analysis. Lesion stages were based on immunohistochemical detection for myelin proteolipid protein (PLP) to detect areas of myelin loss and expression of human leukocyte antigen DR (HLA-DR) (Kipp *et al.* 2012). Active lesions (*n* = 5) were characterized by a focal area of myelin loss filled with myelin-laden ‘foamy’ macrophages. Blocks containing normal appearing white matter (NAWM, *n* = 4) from MS cases and blocks (*n* = 4) containing white matter from control cases were collected.

### Protein Extraction

Samples were placed in extraction buffer, containing RIPA buffer (Sigma, R0278-500ML) supplemented with 1% fos-cholin 16 (Anatrace, F316S-1GM) and protease inhibitor cocktail (Roche, 0469313001). Total protein mixture was then lysed using a crusher during 4 successive runs of 10 s, velocity 4, on ice (IAK, T 10 Standard ULTRA-TURRAX). Lysates were incubated for 2 h at 25 °C with gentle agitation (120 rpm) and were centrifuged 10 min at 10,000 ×*g*. Supernatants corresponding to the soluble fraction and pellets corresponding to the insoluble fraction further resuspended in extraction buffer, were aliquoted. Non-centrifuged protein extracts were also used as “total fraction”. Total protein amount from each fraction was evaluated using the Protein Assay Reagent kit (Pierce, 1,861,426).

### Deglycosylation

Fifty micrograms of total protein extract was deglycosylated using Protein Deglycosylation Kit from NEB. 5 μL of denaturing buffer (B6045S) was added to 40 μL of diluted protein extract and incubated 10 min at 70 °C. Then, after cooling on ice, 5 μL of enzyme mix (P6044S) was added and incubated for 16 h at 37 °C.

### Protein Size Separation Using AMICON Columns

Five-hundred microliters of protein extract was loaded in AMICON Ultra-0.5 100 K device (Merck-Millipore). High molecular weight proteins (> 120 kDa) were enriched on column filter by 30 min centrifugation at 14,000 ×*g*. Filtrate (proteins < 120 kDa, 450 µL) and concentrate (> 120 kDa, 50 µL) were stored separately at –80 °C.

### Automated Capillary Western Blot and Antibodies

Western blots were performed on WES device using Simple Western technology, an automated capillary-based size sorting and immunolabeling system (ProteinSimple™). All procedures were performed with manufacturer's reagents according to their manual. Briefly, diluted protein lysate was mixed with fluorescent master mix and heated at 95 °C for 5 min. The samples (2 mg/mL for HEK lysates or 300 µg/mL for brain samples), blocking reagent, wash buffer, primary antibodies, streptavidin or secondary HRP-coupled antibodies, and chemiluminescent substrate were dispensed into microplate. Protein samples were loaded into individual capillaries on a 25 capillary cartridge (12–230 kDa or 66–440 kDa separation matrix) provided by the manufacturer. Protein separation and immunodetection was performed automatically on individual capillaries using default settings. Primary antibodies, GN_mAb_Env01-biotin (20 µg/mL) or GN_mAb_Env04 (35 µg/mL) were raised against HERV-W ENV protein produced by clones from MS retroviral particles (Komurian-Pradel *et al.* 1999; Perron *et al.* 2001). These monoclonals were shown to display high specificity and affinity for pHERV-W ENV (MSRV-ENV) in various studies from independent groups (Perron *et al.* 2005; 2012; 2013; Kremer *et al.* 2013; 2015; 2019b; Curtin *et al.* 2015; van Horssen *et al.* 2016; Levet *et al.* 2017; Charvet *et al.* 2018) GN_mAb_ENV 16 is directed against syncytin-1 protein (Blond *et al.* 1999) and was selected for its high affinity. The secondary antibody used for the luminometric detection was an anti-mouse secondary antibody from ProteinSimple, USA (cat. # 042-205) as part of their “Anti-Mouse Detection Module” (ProteinSimple, cat. # DM-002). In order to decrease background signal in highly concentrated samples, GN_mAb_Env01 antibody was biotinylated (Biotem, France) and revealed using streptavidin-HRP from ProteinSimple (Biotin detection module ref. 043-459). The theoretical MW of the hexamer based on non-glycosylated monomer MW was about 360 kDa. However, experimental conditions and concentrated samples may yield an enlarged detection range around the calculated MW value corresponding to steric hindrance in capillaries filled with polymer matrix. Consequently, we measured the global signal containing the target hexamer of interest (200–500 kDa, with electrophoregram peak about 350–400 kDa). WES device was associated with Compass software for device settings and raw data recording (ProteinSimple/Biotechne).

### Immunofluorescence

HEK-293 T transfected cells were cultured on 8 wells Lab-Tek culture slides (Nunc, C7182). 15 h or 48 h after transfection, cells were washed once with 200 µL of Phosphate-Buffered Saline 1× (prepared from PBS 10× pH 7.4 Gibco, 70,011–036) and fixed in 4% paraformaldehyde solution (Alfa Aesar, J61899) for 15 min. Cells were further washed three times with 200 µL of PBS 1× and permeabilized with 200 µL of PBS 1× supplemented with 0.2% Tween 20 (Sigma, P7949) for 15 min. After 30 min of incubation in 200 µL of blocking solution made with PBS 1×, 0.2% Tween 20 and 2.5% horse serum (ATCC, 30–2040), cells were incubated with 50 µg/mL of primary antibodies GN_mAb_Env01 diluted in blocking solution for 1 h. After 3 washes with 200 µL of PBS 1×, cells were incubated for 1 h with 4 µg/mL Alexa Fluor 488 goat anti-mouse IgG antibody (ThermoFisher, A11029) diluted in blocking solution. After 3 washes with 200 µL of PBS 1×, cells were counterstained with DAPI/anti-fade mounting medium (Vectashield, H-1500). Microscopy was performed with a Zeiss AXIO Scope A.1 microscope, equipped with Zeiss AxioCam MRm camera. Composite images were created using ImageJ software.

### ELISA

Sulfatides (S1006-5MG), monosialoganglioside (GM1, G7641-5MG), disialoganglioside (GD1a, G2392-5MG) and trisialoganglioside (GT1b, G3767-5MG) were purchased from Sigma-Aldrich (Saint Quentin Fallavier, France). The glycolipids were dissolved in methanol/water 1:1 (by volume). Glycolipid suspensions were heated at 37 °C and sonicated during 20–30 s to obtain a clear solution. Fifty microliters containing the required amount was put into each well of 96-well Elisa plates. The plates were left under a ventilated hood for 25 h to coat the wells. After washing three times with phosphate-buffered saline containing 0.05% Tween 20 (PBS-T), the wells were filled with 200 µL of 5% defatted milk in PBS for 2 h. The defatted milk solution was removed and the wells were washed with PBS-T. One-hundred microliters of PBS containing 50 ng rEnv was added in each well and incubated for 1 h at 37 °C. The wells were washed with PBS-T three times and filled with 1 µg of GN_mAb_Env01 in 100 µL PBS and incubated for 3 h at 37 °C. After washing three times with PBS-T, the wells were filled with 5 µg of biotinylated sheep anti-mouse IgG (Sigma-Aldrich) in 100 µL PBS-T and incubated for 1 h at 37 °C. The wells were washed three times with PBS-T, filled with 5 µg of streptavidin–horseradish peroxidase complex (Sigma-Aldrich) and incubated at 37 °C for 1 h. Then, the wells were washed extensively with PBS-T and the bound peroxidase was visualized under stirring with 200 µL of 4-chloro-naphtol (Sigma-Aldrich) 0.2% in methanol–water 7:3 (v/v). After 15 min incubation, the plates were read at 630 nm. Results were expressed in corrected optic density (OD: values of untreated condition were subtracted from corresponding other conditions).

### Statistical Analysis

Unless otherwise indicated, student's t-test was used for group comparison when data passed the normality test, otherwise non-parametric Mann–Whitney rank sum test was used. *P* values < 0.05 were considered significant. Statistical analyses were performed with Prism 7 (GraphPad Software) for calculations and data plot.

## Results

### Differences between Physiological and Pathogenic HERV-W Envelope Glycoproteins

**Fig. 1:**
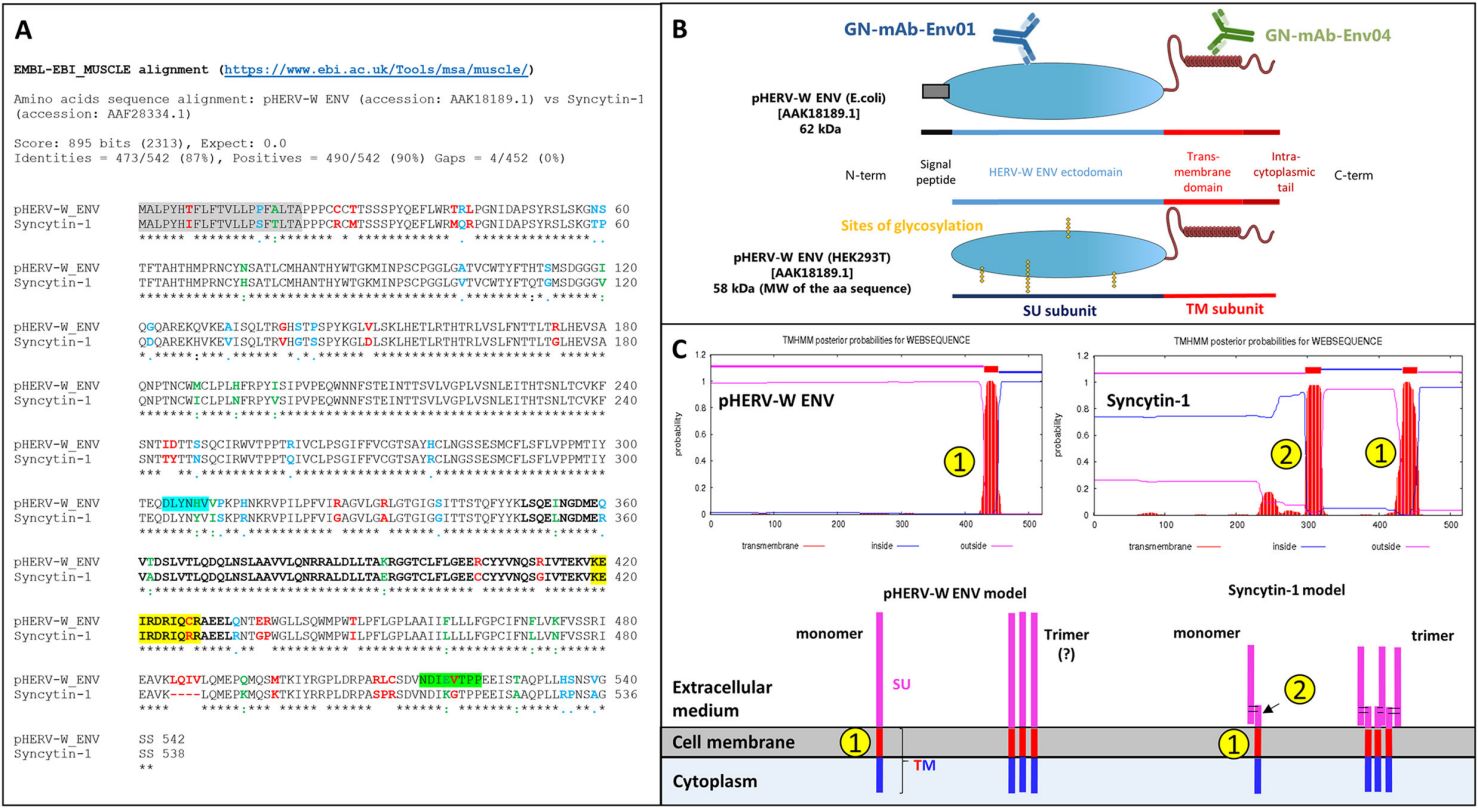
pHERV-W ENV sequence characteristics versus syncytin-1. **A** Aligned amino acid sequences of pHERV-W ENV (GenBank accession no. AAK18189.1) and syncytin-1 (*ERVWE1*-encoded; GenBank accession no. AAF28334.1). Signal peptides are highlighted in grey. The epitope specifically recognized by GN_mAb_Env01 is highlighted in blue. The epitope specifically recognized by GN_mAb_Env04 is highlighted in green. Letters in bold delineate the sequence corresponding to the site of trimerization of retroviral envelope glycoproteins. Amino-acids highlighted in yellow delineate the sequence corresponding to site of cleavage by furin enzyme in retroviral envelope glycoproteins. “Asterisks, black letter”: positions that have a conserved residue; “colons, green letter”: conservation between nucleotides with strongly similar properties; “dots, blue letter”: conservation between nucleotides with weakly similar properties; “space, red letter” positions that have a non-homologous residues with divergent properties of different encoded amino-acids. **B** *E.coli* recombinant pHERV-W ENV protein comprising the signal peptide and glycosylated recombinant protein from transfected human cells (HEK293T cells) with cleaved signal peptide. The localization of epitopes targeted by specific antibodies is illustrated on the *E. coli* protein schematic representation. **C** Predictive study of transmembrane domains indicated the pHERV-W ENV and syncytin-1 transmembrane spanning domains, identified by numbers in yellow dots. Number one corresponds to the domain allowing the anchoring in host cell membrane and number two illustrates the hydrophobic fusion peptide involved in fusion with other cell membranes. Extracellular domains are represented by pink lines, membrane-spanning domains by red lines and intracellular domain by blue ones.

An HERV-W envelope gene with an open reading frame (ORF), first named “MSRV-env”, was described in cDNA from purified virion-like particles in MS cell cultures (Perron *et al.* 1997b, 2001). “MSRV” sequences led to the identification of HERV-W multicopy family comprising the HERV-W/7q copy encoding syncytin-1 (*ERVWE1* locus). Syncytin is an HERV-W envelope that acquired physiological function through evolution. Its expression, restricted in time and space under the control of progesterone via TGF-beta pathway, plays a crucial role during placenta development diverting the fusogenic properties of this envelope glycoprotein to promote cell-to-cell fusions of cytotrophoblasts into syncytiotrophoblasts bridging maternal and embryonic tissues (Strick *et al.* 2007; Noorali *et al.* 2009). Syncytin-1 is composed of surface subunit (SU) and transmembrane subunit (TM), separated by a furin cleavage site (Cheynet *et al.* 2005). After cleavage, the two subunits form a heterodimer linked by a disulfide bond between C_186_XXC_189_ in SU and C_398_XXXXXXC_405_C_406_ in TM, as in D-type (BaEV and MPMV) and C-type (MoMLV and HTLV) exogenous retroviruses (Cheynet *et al.* 2005) (Fig. 1, Supplementary Figure S2).

Apart from deletions or stop codons, known HERV-W *ENV* sequences other than *ERVWE1* do not encode proteins but share common sequence features with “MSRV-env” sequences. The present study will generically refer to them as “HERV-W *ENV*”, according to the present nomenclature. Corresponding full-length protein has been shown to be involved in the pathogenesis of MS (Kremer *et al.* 2019a) and to play a role in type 1 diabetes (T1D) (Levet *et al.* 2017), but could not already be attributed to specific chromosomal locus or loci within this multicopy family. It will therefore be referred to as “pathogenic HERV-W ENV” (pHERV-W ENV). HERV genomic heterogeneity among human genomes (Thomas *et al.* 2018), probable recombination or rearrangements during pathogenic processes (Kremer *et al.* 2019b) and increased DNA copy number in certain cells (Perron *et al.* 2012; Garcia-Montojo *et al.* 2013), also called for a generic name for this protein with biochemical characteristics diverging from those of syncytin-1.

Syncytin-1 and pHERV-W ENV amino-acid sequences present an identity of 87% (Fig. 1A). Both proteins may be secreted due to the presence of a 20 amino acids (aa) signal peptide. The surface (SU) and transmembrane (TM) subunits are well conserved, including the trimerization site (Fig. 1A; Supplementary Figure S2). However, mutations in the furin cleavage site (R/H_314_NKR) and in the CXXXXXXCC (C/R_405_) motif for disulfide bond formation in pHERV-W ENV indicate that structural organizations should be very different between these two proteins (Gimenez 2007; Grandi and Tramontano 2018a). Indeed, syncytium formation was observed in cultures of cells transfected with plasmids expressing syncytin-1 (GenBank: AF072506.2), but not with plasmids expressing pHERV-W ENV (GenBank: AF331500.1), thereby confirming the loss fusogenic activity (Supplementary Figure S2).

Two mouse monoclonal antibodies with specific pHERV-W ENV epitopes comprising divergent sequences from syncytin-1 were selected: GN_mAb_Env01 targeting the SU subunit and GN_mAb_Env04 targeting the intracytoplasmic part of the TM subunit (Fig. 1B). As shown with the alignment of their amino acid sequences (Fig. 1A; Supplementary Figure S2), pHERV-W ENV and syncytin-1 show 90% homology when including chemically analogous amino acids, but syncytin-1 shows a gap of 4 aa in the C-terminal region (aa385 to aa388, 12 nucleotides in *ERVWE1* gene). This represents a molecular signature of syncytin-1. To date, all other identified HERV-W copies revealed homologous to the prototypic MSRV-*env* nucleotide sequence (Perron *et al.* 1997b; Perron *et al.* 2001; Komurian-Pradel *et al.* 1999), i.e. they do not lack these 12 nucleotides.

A predictive study of the transmembrane domain reveals two membrane spanning domains in syncytin-1 sequences, as previously described (Gong *et al.* 2005; Grandi and Tramontano 2018a). The first one, located around aa300, corresponds to the fusion peptide, unmasked during the pre- to post-fusion conformational transition, inducing cell–cell membrane fusion. The second one, located around aa430, is a regular anchoring transmembrane domain (Ruigrok *et al.* 2019). Interestingly, pHERV-W ENV has only one membrane-spanning site that corresponds to the site of cell membrane anchoring domain. The additional loss of functional fusion peptide domain of pHERV-W ENV reinforces the behavioral differences between the physiological syncytin-1 and the pathogenic pHERV-W ENV envelope glycoproteins (Fig. 1C). Despite a high degree of sequence conservation, both HERV-W envelopes present a quite different panel of functional domains, e.g., involving furin cleavage and fusion machinery. Consequently, each protein will neither have the same behavior nor the same biological activities.

### Self-Assembly Properties of pHERV-W ENV

pHERV-W ENV is a globally hydrophobic protein with an isoelectric pH (pHi) above 9, implying electropositivity of its peptide structure at physiological (neutral) pH.

**Fig. 2:**
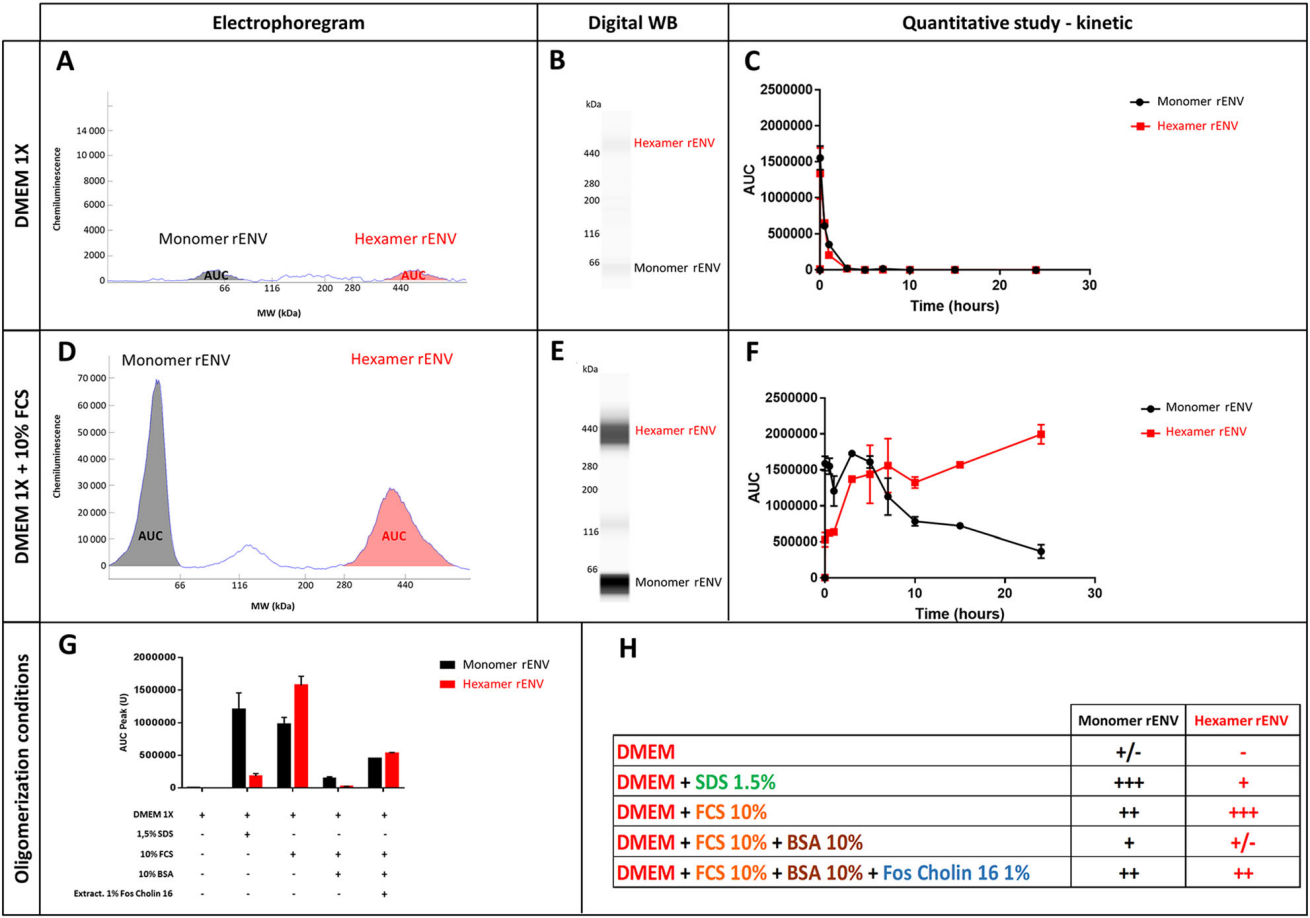
Recombinant pHERV-W ENV oligomerization. Automated capillary Western blot technology (Simple Western®) was used for characterizing pHERV-W ENV antigen produced in* E. coli* expressing system (rENV). pHERV-W ENV detection was performed with GN_mAb_Env01 antibody. This highly sensitive technology provided different types of information: migration electrophoregrams (**A, D**), digital Western blots (**B, E**) and quantitative interpretation based on calculation of the area under curve -AUC- (**C, F** and **G**). Here, the self-assembly properties of pHERV-W ENV were assessed in basic DMEM or DMEM media with 10% FBS (**A–F**). Kinetics of oligomerization were performed within 24 h of incubation, supernatants were harvested at different time-points and each form of pHERV-W ENV was quantified (**C, F**). pHERV-W ENV oligomerization characteristics after overnight (ON) incubation at 37 °C in presence of 1.5% SDS, 10% FBS, 10% FBS + 10% BSA extracted without fos-cholin 16, or with fos-cholin 16 when mentioned, are documented in **G** and **H**. All experiments were repeated 6 times.

Of note, this induces electrostatic interaction with plastic surfaces and a rapid loss of recombinant protein when diluted in media such as PBS1X (data not shown) or DMEM 1X (Fig. 2A–2C). Kinetics of disappearance of the protein from the aqueous medium revealed a 50% loss after 10 min of incubation at 37 °C in DMEM and undetectable protein in the solution after 3 h (Fig. 2C). This electrostatic capture of pHERV-W ENV was prevented with 10% fetal bovine serum (FBS), which blocks plastic charges with serum proteins but may also offer alternative electrostatic bonds in solution for pHERV-W ENV at neutral pH (Fig. 2D–2F).

We further analyzed the immunodetection profile of the non-glycosylated recombinant protein by capillary electrophoresis (WES, Bio-Techne/ProteinSimple, USA) under denaturing conditions. This evidenced that pHERV-W ENV is detected under denaturation-resistant oligomeric forms: a monomer (62 kDa), a high MW oligomer (380–440 kDa) corresponding to an hexamer and a minor form (120 kDa) corresponding to a dimer (Fig. 2D–2F). The latter form was further shown to result from partial degradation of the hexamer in denaturing conditions (Supplementary Figure S3). Repeated analyses (*n *= 6) confirmed that self-assembly of pHERV-W ENV yields an hexameric structure, fitting with the MW of six monomers within the limits of resolution of the separation matrix in capillaries. Kinetics studies with recombinant monomers in 10% FBS DMEM 1X evidenced a thermodynamic process with a decrease in monomers over time stabilizing after a parallel increase of hexamers representing the dominant form (Fig. 2F). Thus, pHERV-W ENV protein can self-assemble under hexameric macromolecular structure in the absence of cells or of other cellular membrane sources, whereas no trimer is detected. This indicates that the trimeric form may not be required for hexameric formation (Fig. 5B).

Further study of electrostatic forces responsible of unbound pHERV-W ENV disappearance in protein-free aqueous media was performed after this oligomeric profile was characterized. 1.5% SDS was added in DMEM medium to neutralize the electropositivity of pHERV-W ENV and, after overnight incubation at 37 °C, the monomer was still present in the medium. However, unlike incubation in DMEM with FBS, it only yielded a low proportion of hexamer. This showed that oligomerization of monomers can be inhibited by SDS. pHERV-W conformation and/or global charge, since modified by SDS, appeared critical for oligomer formation (Fig. 2G, 2H).

Since previous observations indicated a loss of pHERV-W ENV detection when spiked in total serum (data not shown), we addressed a possible interaction with albumin which represents 50% of serum proteins. Beyond its global negative charge like other plasma proteins at physiological pH, albumin efficiently binds hydrophobic moieties of various molecules. Sufficient addition of bovine serum albumin (BSA) to DMEM with 10% FBS inhibited the detection of pHERV-W ENV in either monomeric or hexameric forms. Results supported a masking role of albumin above a concentration threshold not reached when using 10% FBS in the medium. This is likely to involve the hydrophobic moiety of pHERV-W ENV protein in hydrophobic interactions with serum albumin (Fatima *et al.* 2017a, 2017b) and suggests an additional type of interaction than previously shown for pHERV-W ENV global electropositive charge. Hypothesizing effective hydrophobic interactions with albumin in solution, we extracted total proteins from this mixture with BSA using 1% fos-cholin 16 diluted in RIPA buffer to disrupt macromolecular complexes formed by both electrostatic and hydrophobic bonds. In the presence of fos-cholin 16, but not in the absence of (data not shown), we could partially rescue pHERV-W ENV detection (Fig. 2G, 2H). This indicated that fos-cholin 16 in RIPA buffer should be used for an efficient extraction of pHERV-W ENV antigens in the following analyses of cultured cells or tissue samples.

### pHERV-W ENV Antigens Characterization in Human Cell Lines

**Fig. 3:**
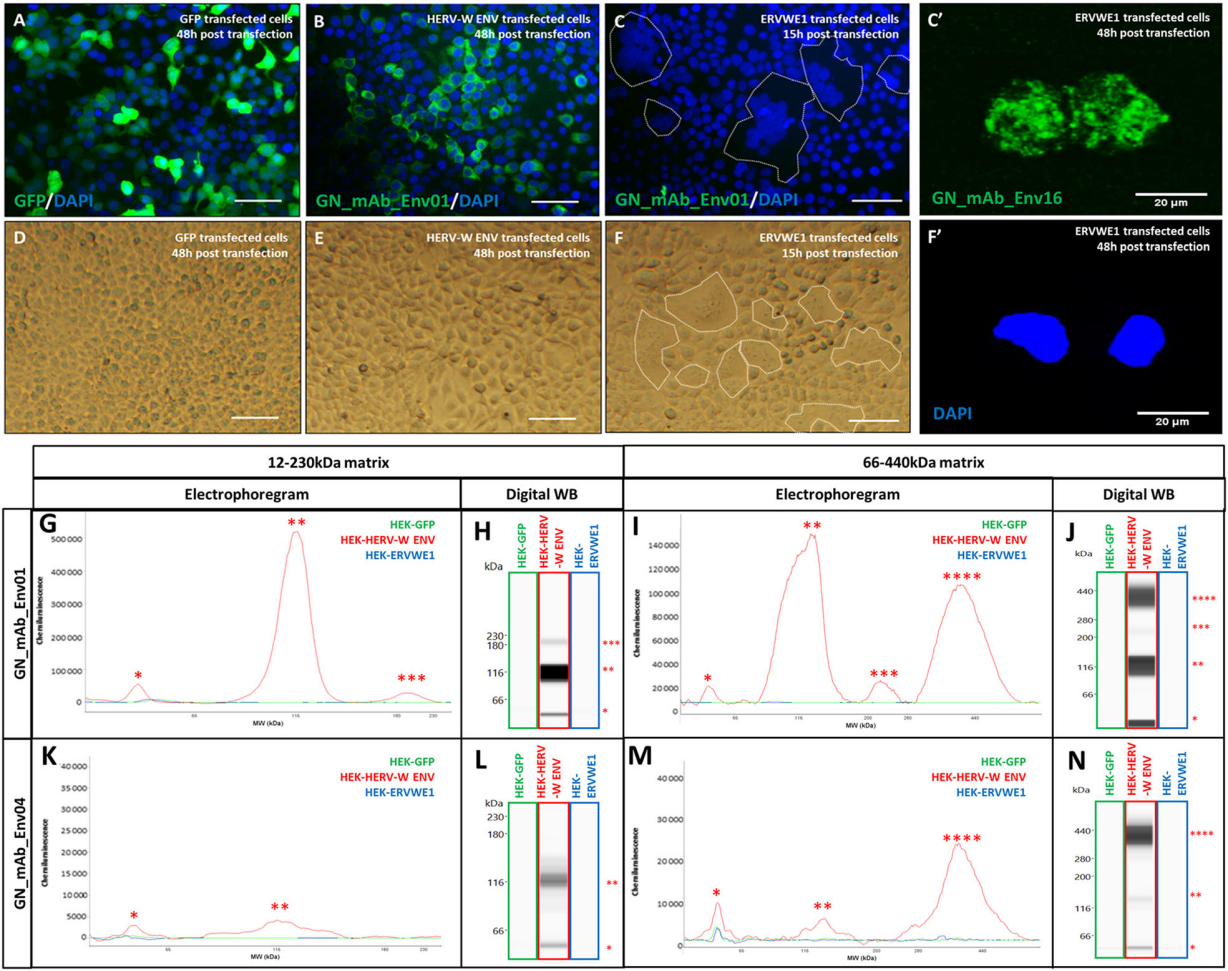
Characterization of pHERV-W ENV antigen expressed in transfected human cells. Immunofluorescence staining with GN_mAb_Env01 in cells transfected with sequences encoding GFP (**A**), pHERV-W ENV (HERV-W *ENV*, **B**) or syncytin-1 (*ERVWE1*, **C**). Immunofluorescence (IF) staining with GN_mAb_Env 16 in cells transfected with sequences encoding syncytin-1 (*ERVWE1*, **C’**). Cell nuclei were stained with DAPI and appeared with blue fluorescence (**A–C; F’**). In **C** and **F**, syncytia formation was shown to be significant at 15 h while preserving the cell layer for IF. Longer delays generated enlarged syncytia shedding from the slides or with fragile membrane structures that impaired cell fixation and detection on slides by IF. Thus, immunolabelling with anti-syncytin antibody at 48 h is shown with GN mAb_Env 16 as with isolated syncytium remaining fixed on the slide after 48 h (**C’**). No immunostaining was observed using GN_mAb_Env01 for GFP or syncytin-1. **D–F** Bright field microscopy pictures of cell cultures transfected with the same plasmids encoding GFP (**D**), pHERV-W ENV (**E**) and syncytin-1 (**F**). Syncytin-1 expression was accompanied by typical formation of syncytia, as delimited by a dotted line (**C, F**) showing grouped DAPI-stained nuclei in the center (**C**). Bar = 50 µm. DAPI staining corresponding to **C’** at 48 h is shown in **F’**. Lysates of same HEK293T cells transfected with same sequences encoding GFP (green panel), pHERV-W ENV (HERV-W ENV, red panel) or syncytin-1 (ERVWE1, blue panel) were analyzed by Simple Western® using GN_mAb_Env01 (**G**–**J**) or GN_mAb_Env04 (**K**–**N**) antibodies. For these analyses, cells could be collected after the same delay, pelleted and lysed in parallel. In order to precisely observe all antigens forms, the immunodetection was performed on 12–230 kDa (**G**–**H** and **K**–**L**) and 66–440 kDa (**I**–**J** and **M**–**N**) size separation matrices. Results are presented as migration electrophoregrams (**G**, **K**, **I**, **M**) and digital Western blot (**H**, **J**, **L**, **N**). *: pHERV-W ENV monomer, **: glycosylated pHERV-W ENV monomer (Cf. also Supplementary Figure S2), ***: pHERV-W ENV trimer, ****: pHERV-W ENV hexamer. All experiments were repeated 6 times.

Recombinant pHERV-W ENV produced in *E. coli *lacks glycosylation and cleavage of the signal peptide. To determine the possible impact of post-translational modifications on pHERV-W ENV oligomerization process, human HEK293T cells were transfected in parallel with genes encoding GFP, syncytin-1 (*ERVWE1*) and pHERV-W ENV (HERV-W *ENV*)*.* Transfection efficiency was assessed using GFP detection in GFP transfected cells (Fig. 3A). Immunofluorescence with GN_mAb_Env01 antibody specifically detected pHERV-W ENV in cells transfected with encoding plasmid (Fig. 3B) and not when transfected with *ERVWE1* sequences (Fig. 3C). Syncytin-1 protein production was confirmed by immunostaining with anti-syncytin monoclonal and syncytia formation in *ERVWE1* transfected cells (Fig. 3C, 3C′, 3F, 3F′). No syncytium was observed in pHERV-W ENV expressing cells, confirming that this envelope glycoprotein has no detectable fusogenic property (Fig. 3B–3E).

The immunochemical profile of glycosylated pHERV-W ENV with cleaved signal peptide, as expressed in transfected human cells was analyzed using the same WES platform with two capillary matrices for low- and high-molecular weights and two pHERV-W ENV specific antibodies (GN_mAb_Env01 and GN_mAb_Env04).

Four antigenic forms of HERV-W ENV were observed in lysates of HERV-W *ENV* transfected cells, using the previously optimized buffer with fos-cholin 16 detergent, at 58 kDa (monomer), 116 kDa (further shown to represent both glycosylated monomers and denaturation-related dimers), 230 kDa (trimer) and 360–440 kDa (hexamer) (Fig. 3G–3N).

### pHERV-W ENV and Syncytin-1 Differing Immunogenicity and Biochemical Properties

Equal quantities of extracted proteins from transfected cells with one of the three plasmids encoding GFP, syncytin-1 or pHERV-W ENV, were analyzed. While showing no specific detection of GFP or of syncytin-1 with GN_mAb_Env01 or GN_mAb_Env04 antibodies, results clearly confirmed detection of pHERV-W ENV by both antibodies (Fig. 3G–3N). Unique pHERV-W ENV detection was also confirmed by immunocytochemistry (Fig. 3B). Detection with GN_mAb_Env01 was much more efficient, which may indicate a rather buried epitope in the conformation of N-term extremity, for GN_mAb_Env04.

In addition, the furin cleavage site between SU and TM regions in pHERV-W ENV was confirmed not to be functional since amino-acid sequences were detected by both monoclonal antibodies targeting either the SU or the TM domains within the same molecular structure. They also correspond to MW of SU+TM monomers or oligomers (Fig. 3G, 3H, 3K, 3L).

### Solubility, Hydrophilicity and Hydrophobicity of Glycosylated pHERV-W ENV Antigen Forms

Because different oligomers may confer diverging properties to (macro-)molecular structures, we analyzed their solubility in aqueous buffers used for protein extraction.

**Fig. 4:**
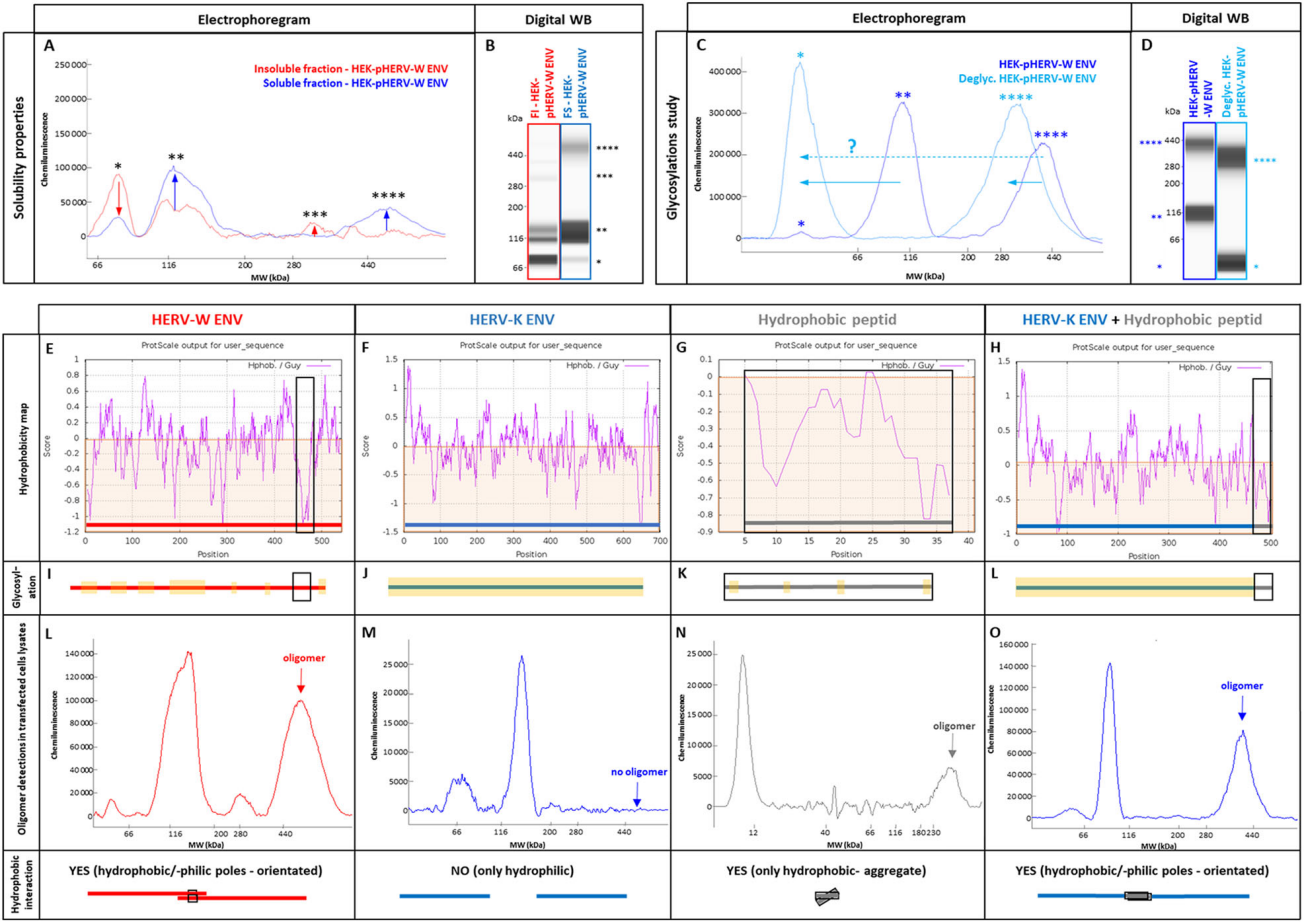
pHERV-W ENV physico-chemical characterization. After RIPA-fos-cholin 16 extraction, transfected HEK293T cell lysates were centrifuged and the soluble fraction (**A, B**, blue panel) was separated from the insoluble fraction (**A, B**, red panel). The soluble fraction containing pHERV-W ENV hexamer was deglycosylated (**C, D**, clear blue panel) and compared to an non-deglycosylated profile of the soluble fraction (**C, D**, darker blue panel). pHERV-W ENV antigen detection was performed using Simple Western® immunoassay and results are presented as migration electrophoregrams (**A, C**) and digital western blot (**B, D**). **A**: arrows indicate which oligomers were enriched or depleted in soluble or insoluble fractions. **C**: arrows highlight the shift in molecular mass after deglycosylation. *pHERV-W ENV monomer, **glycosylated pHERV-W ENV monomer (Supplementary Figure S3), ***pHERV-W ENV trimer, ****pHERV-W ENV hexamer. All experiments were repeated 6 times. Hydrophobic amino acid mapping was performed using “Guy’s hydrophobic score calculation” along the peptide sequence of pHERV-W ENV (**E, I, L**; column with red panel), HERV-K ENV (**F, J, M**; column with blue panel), a synthetic 37aa highly hydrophobic peptide (HP) (**G, K, N**; column with grey panel) and the fusion protein HERV-K ENV-HP (**H, L, O**; column with blue + grey panel). Guy’s score > 0 reveals hydrophilic amino acids and residues obtaining a score < 0 are hydrophobic (brown areas). Hydrophobic domains are highlighted on each graph using a black square. Hydropathic index, revealing the global hydrophobicity of protein, is calculated for each construction. As glycosylations can counteract hydrophobicity, a schematic map of predictive glycosylation sites highlighted in yellow is presented (**I, J, K, L**). Electrophoregrams obtained by Simple Western® with lysates of transfected HEK293T cells were analyzed in the high molecular weight part of the separation matrix. pHERV-W ENV was detected with GN_mAb_Env01 antibody (**E, I, L**; column with red panel), HERV-K ENV and HERV-K ENV-HP (**G–O**; column with blue panel) were detected with GN_mAb_Env-K01 antibody, HP was detected using an anti-FLAG tag polyclonal antibody (**G, K, N**; column with grey panel). The ability of each construct to form hydrophobic interactions and to link two monomers via their hydrophobic domains, labeled “YES”, is schematically represented at the bottom line of the figure by black squares.

**Fig. 5:**
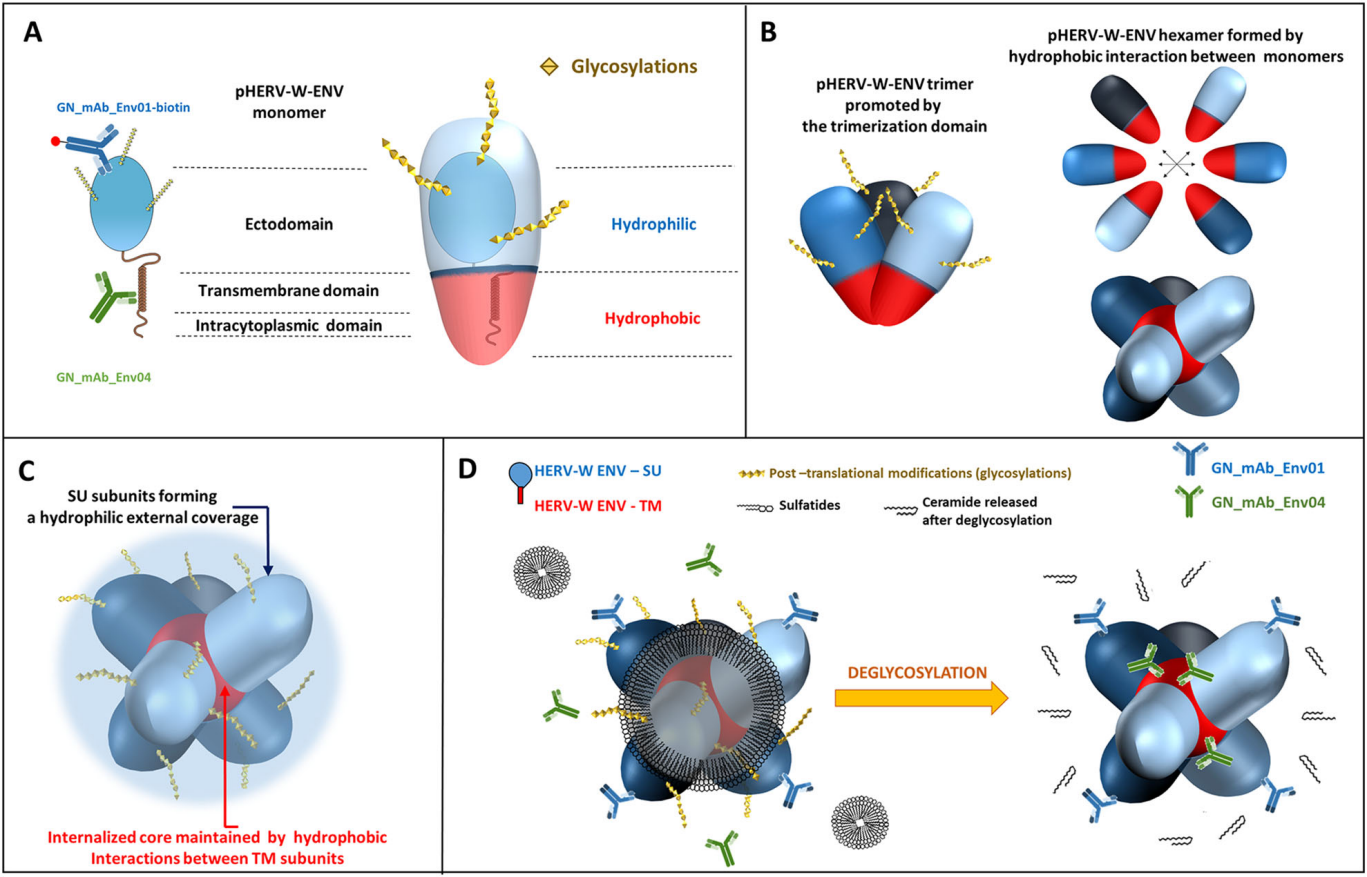
Schematic models for pHERV-W ENV antigens. **A** Schematic representation of pHERV-W ENV monomer with GN_mAb_Env01 and GN_mAb_Env04 antibodies binding sites. Blue and red overlays represent hydrophilic and hydrophobic domains of pHERV-W ENV, respectively. Yellow stars represent glycosylations. **B** pHERV-W ENV trimer formation driven by retroviral envelope trimerization domain conserved on pHERV-W ENV. pHERV-W ENV hydrophobic domains are still exposed despite trimer organization and retain the ability to induce hydrophobic interactions for hexameric formation, which does not reveal accurate but driven by monomers, as results showed. **C** pHERV-W ENV hexamer 3D-like representation. **D** Macrostructural representation showing that GN_mAb_Env01 epitope is accessible on the exposed hydrophilic ectodomain, contrary to GN_mAb_Env04 epitope buried in the hydrophobic core and masked by a layer of sulfatides. Deglycosylation induces sulfatides removal (by oside heads degradation) in addition of the opening of the deglycosylated protein structure, thereby making GN_mAb_Env04 epitope accessible to the antibody.

Glycosylated monomers and dimeric forms generated by the degradation of hexamers in denaturing conditions were partly detected in the soluble fraction, unlike with the non-glycosylated recombinant. This is consistent with known increased solubility of hydrophobic proteins with glycosylations. The trimer was only detected in the insoluble fraction, consistent with the small amount of membrane-inserted trimer that remained totally trapped in pelleted cell debris (data not shown). However, the hexamer was uniquely detected in the soluble fraction (Fig. 4A, 4B), suggesting that this hexameric organization conferred hydrosolubility to pHERV-W ENV antigen. This is consistent with an external hydrophilic ring masking an internalized hydrophobic core, thereby stabilizing the structure in aqueous media (Fig. 5C).

### Influence of Glycosylations

As shown in Fig. 4C, 4D, enzymatic deglycosylation of extracted proteins induced a mass shift of the 116 kDa form to 58 kDa, suggesting that the 116 kDa form corresponded to the glycosylated form of pHERV-W ENV monomer with a non-glycosylated monomer at 58 kDa, which is the predicted MW of the matured pHERV-W ENV protein after the cleavage of the signal peptide (Cf. Figure 1). Nonetheless, it was surprising to observe that the antigen detected around 116 kDa may also correspond to a dimer of the non-glycosylated protein. Indeed, further investigation revealed that a strong denaturing treatment induced the formation of a “double peak” at ~120 kDa, paralleling a strong decrease of the hexamer signal (Supplementary Figure S3A–S3D). Interestingly, the deglycosylation of the strongly denatured protein mix induced a mass shift only for one of the two peaks around ~120 kDa, indicating that the deglycosylation-sensitive form corresponded to the glycosylated monomer when the deglycosylation-insensitive form corresponded to a dimer of unglycosylated pHERV-W ENV generated by the denaturation of pHERV-W ENV hexamers (Supplementary Figure S3E–S3H). This also indicates that unglycosylated forms may be produced in eukaryotic cells, at least in HEK-293 T cell line. Such co-expression of glycosylated and unglycosylated forms of the same protein is known to occur, also representing particular pathogenic signatures in prion diseases (Ironside and Head 2004; Xanthopoulos *et al.* 2009).

Concerning the forms detected at 280 kDa and around 400 kDa, they correspond respectively to the trimeric and to the hexameric forms of pHERV-W ENV. The mass shift of the hexameric peak observed in the soluble fraction after deglycosylation appears consistent despite the lower resolution in higher MW: from about 400 kDa to about 350 kDa.

### Hydrophobic Interactions Trigger HERV-W ENV Hexamer Formation

Both pHERV-W ENV and syncytin-1 have a consensus trimerization site (aa350 to aa432). Syncytin-1 trimerization mechanism was described (Gong *et al.* 2005). Critical amino-acids involved in the putative coiled-coil association are conserved in pHERV-W ENV. Two monomers of syncytin-1 are able to trigger bonds in an antiparallel manner. L386 and L375 interact with I413 and I421, respectively, trough hydrophobic interactions. T367 and R383 create hydrogen bonds with R433 (Q433 in pHERV-W ENV) and Q410, respectively. pHERV-W ENV also yields trimeric organization (Fig. 3I, 3J). Beyond trimer formation via the trimerization site, hydrophobic interactions between uncleaved TM moieties of two trimers may allow the formation of an hexamer stabilized by the hydrophobic core formed by bound TM units.

pHERV-W ENV is a globally hydrophobic protein (hydropathic index = –0.0679; close to zero, meaning poorly hydrophilic). A detailed analysis with the hydrophobicity map based on Guy’s calculation table, which attributes a score to each amino-acid reflecting its hydrophobicity, revealed the presence of a highly hydrophobic site located on a large sequence (aa420 to aa480, Fig. 4E). Predictions of glycosylation sites showed that large parts of the protein containing the most hydrophobic sequences are not glycosylated, therefore constantly remaining hydrophobic. In aqueous or extracellular media, thermodynamics of monomers/trimers may thus create strong hydrophobic bonds burying TM domains in a spheric structure surrounded by the SU polar part outside the structure. This structure fits with the observed MW of hexamers in presented analyses (Fig. 4 HERV-W ENV/red panel E, I, L and Fig. 5C).

To confirm this hypothesis based on hydrophobic interactions leading to the conformational organization that would explain observations made with pHERV-W ENV antigen, a much more soluble HERV envelope glycoprotein (hydropathic index = −0.2424) from another HERV family was studied: HERV-K ENV (HERV-K113). HERV-K ENV hydrophobicity map showed no large hydrophobic domain like in HERV-W ENV (Fig. 4F). Moreover, glycosylation site predictions indicated potential glycosylations all over the protein, which also predicted an increased solubility provided by glycans. Interestingly, WES analysis of HERV-K ENV with specific monoclonal antibodies only showed monomers, without oligomer (Fig. 4M). A construct encoding a very hydrophobic peptide (HP) almost composed of hydrophobic amino-acids was also designed (Cf. Supplementary Figure S1). HP hydrophobicity map (Fig. 4G) and hydropathy index calculation (hydropathic index =  + 0.0854; positive, meaning globally hydrophobic) confirmed its strong hydrophobicity. Glycosylation sites were barely predicted and the capability of this peptide to aggregate in solution was confirmed by WES profile (Fig. 4N).

To mimic the hydrophobic domain of pHERV-W ENV in HERV-K ENV, we used a plasmid encoding a fusion protein with HP peptide linked to the SU part of HERV-K ENV (SU-HP), replacing the initial TM part, in order to generate a highly hydrophobic domain on its C-term extremity (Fig. 4H). WES analysis of (HERV-K ENV) SU-HP revealed that HP addition conferred oligomerization properties to HERV-K ENV (Fig. 4O). This observation thereby confirmed our hypothesis: pHERV-W ENV hexamer formation can be triggered by the highly hydrophobic TM domain of pHERV-W ENV, since not cleaved in the absence of a functional furin site. Thus, biochemical characteristics of pHERV-W ENV appear quite original, compared to HERV-K envelope antigen, or to syncytin-1 that represents another HERV-W envelope protein.

### Characterization of a Soluble High Molecular Weight pHERV-W ENV Antigen in MS Brain Lesions

pHERV-W ENV was detected by immunohistology in all MS post-mortem brains analyzed to date, quite selectively associated with areas of still ongoing demyelination and neurodegeneration in post-mortem tissue (Kremer *et al.* 2013, 2019a; van Horssen *et al.* 2016). Producing cells appeared to be dominantly microglia and non-CD3 cells of lymphoid cuffs in the vicinity of actively demyelinating areas (van Horssen *et al.* 2016; Kremer *et al.* 2019a). To determine the molecular profile and the solubility of pHERV-W ENV antigen in MS brains, we have analyzed frozen tissue blocks from MS active lesions, from MS normal appearing white matter (NAWM) and from non-MS brains white matter (WM), with the same capillary immunoelectrophoretic approach (WES) as previously used for proteins expressed by reference clones.

**Fig. 6:**
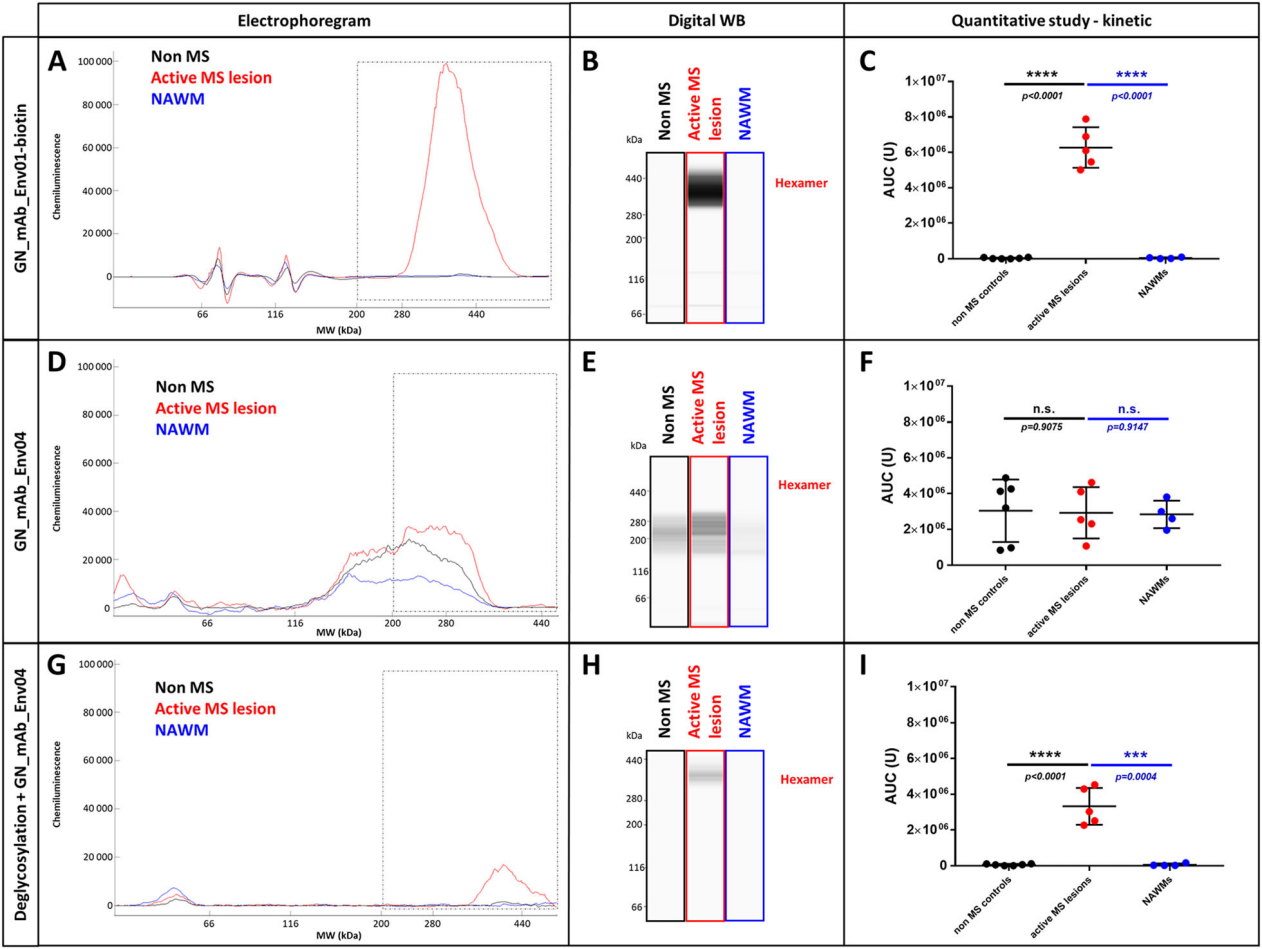
pHERV-W ENV soluble and stable hexamer detection as a hallmark of MS active lesions. Soluble fractions from frozen brain white matter blocks were prepared by protein extraction and enrichment of kDa ≥ 120 kDa MW proteins (AMICON column). Simple Western® profiles of pHERV-W ENV antigens were prepared as in previous analyses with fos-cholin 16 under denaturing conditions, and immunolabelled with GN_mAb_Env01-biotin (**A–C**) or GN_mAb_Env04 (**D–I**) antibodies. Non-MS controls brain white matter (black panels, *n* = 6), active MS lesion (red panel, *n* 5) and normal appearing white matter (NAWM) from MS brain (blue panel,* n* = 4). LLysates of same HEK293T cells transfected with same sequences encoding GFP (green panel), pHERV-W ENV (HERV-W ENV, red panel) or syncytin-1 (*ERVWE1*, blue panel) were analyzed by Simple Western® using GN_mAb_Env01 (**G–J**) or GN_mAb_Env04 (**K–N**) antibodies. For these analyses, cells could be collected after the same delay, pelleted and lysed in parallel. In order to precisely observe all antigens forms, the immunodetection was performed on 12–230 kDa (**G–H** and **K–L**) and 66–440 kDa (**I–J** and **M–N**) size separation matrices. Results are presented as migration electrophoregrams (**G, K, I, M**) and digital Western blot (**H, J, L, N**). *pHERV-W ENV monomer, **glycosylated pHERV-W ENV monomer (Cf. also Supplementary Fig. 2), ***pHERV-W ENV trimer, ****pHERV-W ENV hexamer. All experiments were repeated 6 times.

First analyses of extracted proteins from brain tissue evidenced a soluble antigen with the apparent MW of pHERV-W ENV hexamer and the specific detection with biotinylated GN_mAb-Env01 antibody (specific for the surface domain-SU, Cf. Figure 1B and Fig. 5A) in all samples from MS active lesions (Fig. 6A–6C). This was not detected from tissue blocks of NAWM nor in non-MS WM. The area under the curve (AUC) from chromatograms between 200 and 440 kDa for each sample confirmed a significant difference between MS active lesions and MS NAWM, still emphasized between MS lesions and non-MS WM.

Surprisingly, GN_mAb_Env04 antibody, specific for the C-term TM domain (Cf. Figure 1B and Fig. 5A) did not detect its pHERV-W ENV C-terminal epitope in non-deglycosylated protein extract from these samples, only yielding noise signal at lower MW (Fig. 6D–6F). This differed from previous results obtained with the antigen expressed in transfected human cells. Consequently, the TM epitope was either not accessible to the antibody in these glycosylated hexameric forms immunodetected by the anti-SU monoclonal, or the TM subunit was absent from the antigen extracted from MS brain lesions despite consistent MW of about 400 kDa.

After deglycosylation of the same soluble protein extracts from all MS active lesions, the detection of pHERV-W ENV hexamer with GN_mAb_Env04 became effective (Fig. 6G–6I). This showed that deglycosylation modified the antigen structure in a way allowing this antibody to gain access to TM domains, i.e., causing an exposition of the corresponding epitope (Fig. 5D). The histogram representing the AUC of deglycosylated pHERV-W ENV soluble hexamer with GN_mAb_Env04, also showed the unique specificity of this detection when quantified in active MS lesions versus all samples from MS NAWM and non-MS WM (Fig. 6F).

Therefore, the pHERV-W ENV soluble hexameric antigen from MS brain displayed a different biochemical feature than the one expressed from transfected cells. The presence of non-cleaved SU-TM monomers within the macromolecular structure was anyhow confirmed after deglycosylation by the two monoclonal antibodies specific for distant SU and TM epitopes.

Importantly, the evidence of a soluble hexameric form and the presence of a complete SU-TM protein without furin cleavage confirmed by monoclonal antibodies targeting distant epitopes with diverging sequences from and not detected in syncytin-1 (Cf. Fig. 1A and Fig. 3), now provide evidence that the pHERV-W ENV antigen expressed in MS brain lesions is not syncytin-1. So, pHERV-W ENV soluble hexamer is likely to represent a hallmark of MS brain lesions and retains previously observed properties maintaining a hexameric structure after denaturation and deglycosylation, though with differences in properties linked to glycosylations and/or glycan residues (Fig. 6G–6I).

### Interaction with Cerebral Sulfatides Reproduces the Specific Biochemical Properties of pHERV-W ENV Hexamer From MS Brain Lesions

As previously described, the hexamer extracted from MS brain lesions was readily detectable using anti-SU GN_mAb_Env01 antibody (Fig. 6A–6C), but not using anti-TM GN_mAb_Env04 antibody in the absence of deglycosylation step (Fig. 6D–6I). However, pHERV-W ENV hexamer produced in transfected HEK293T cells did not require deglycosylation for immunodetection using the anti-TM antibody (Fig. 3M, 3N). This observation raised the question of a structural difference between the hexamer produced in human embryonic kidney (HEK) cell line culture and the one produced in MS brain lesions, involving deglycosylation-sensitive molecular structures.

Brain parenchyma contains a high amount of lipids (Naudi *et al.* 2015) and the physico-chemical characteristics of pHERV-W ENV hexamer (hydrophobic core, electropositivity, glycosylations) should allow brain lipids to interact with this soluble antigen especially when released from degraded myelin in MS lesions. In glycolipids, fatty acid and ceramide tails may be attracted by hexamers hydrophobic core while the oside head can interact with electropositive pHERV-W ENV amino acids. Moreover, the glycan moiety of glycolipids can be cleaved from the lipid moiety by the enzyme cocktail used in our deglycosylation step, thereby impacting their eventual association with pHERV-W ENV.

**Fig. 7:**
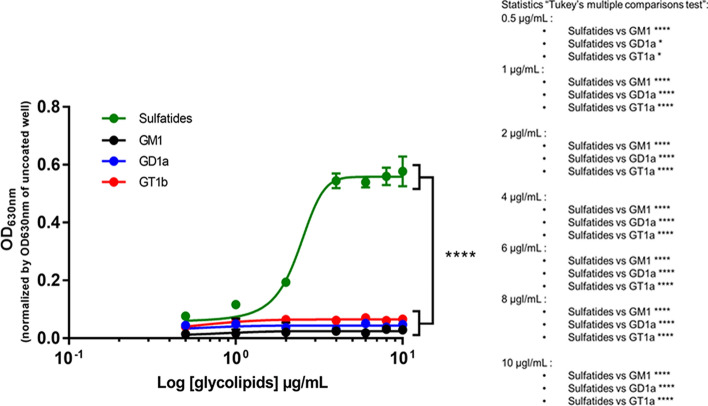
Sulfatides can interact with pHERV-W ENV monomer. Interaction between the purified monomeric recombinant pHERV-W ENV protein and glycolipids was assessed by ELISA. Sulfatides (green curve), monosialoganglioside (GM1, black curve), disialoganglioside (GD1a, blue curve) and trisialoganglioside (GT1b, red curve) were coated on ELISA plate and monomeric pHERV-W ENV was added as a potential ligand. Revelation was performed using GN_mAb_Env01 as primary antibody. The revelation with 4-chloro-naphthol was read at 630 nm. The OD630nm value observed for corresponding wells without coating was subtracted from the presented OD630 nm values. Statistical analysis: Tukey’s multiple comparison test (*P < 0.01, **P < 0.05, ***P < 0,001; ****P < 0.0001)*.* Each experiment was repeated 3 times.

To assess the ability of glycolipids to interact with pHERV-W ENV, we performed an ELISA assay using plates coated with glycolipids. Among the glycolipids tested (sulfatides, monosialoganglioside, disialoganglioside and trisialoganglioside), only sulfatides allowed a significant capture of the recombinant protein pHERV-W ENV, detected with the antibody GN_mAb_Env01 (Fig. 7).

**Fig. 8:**
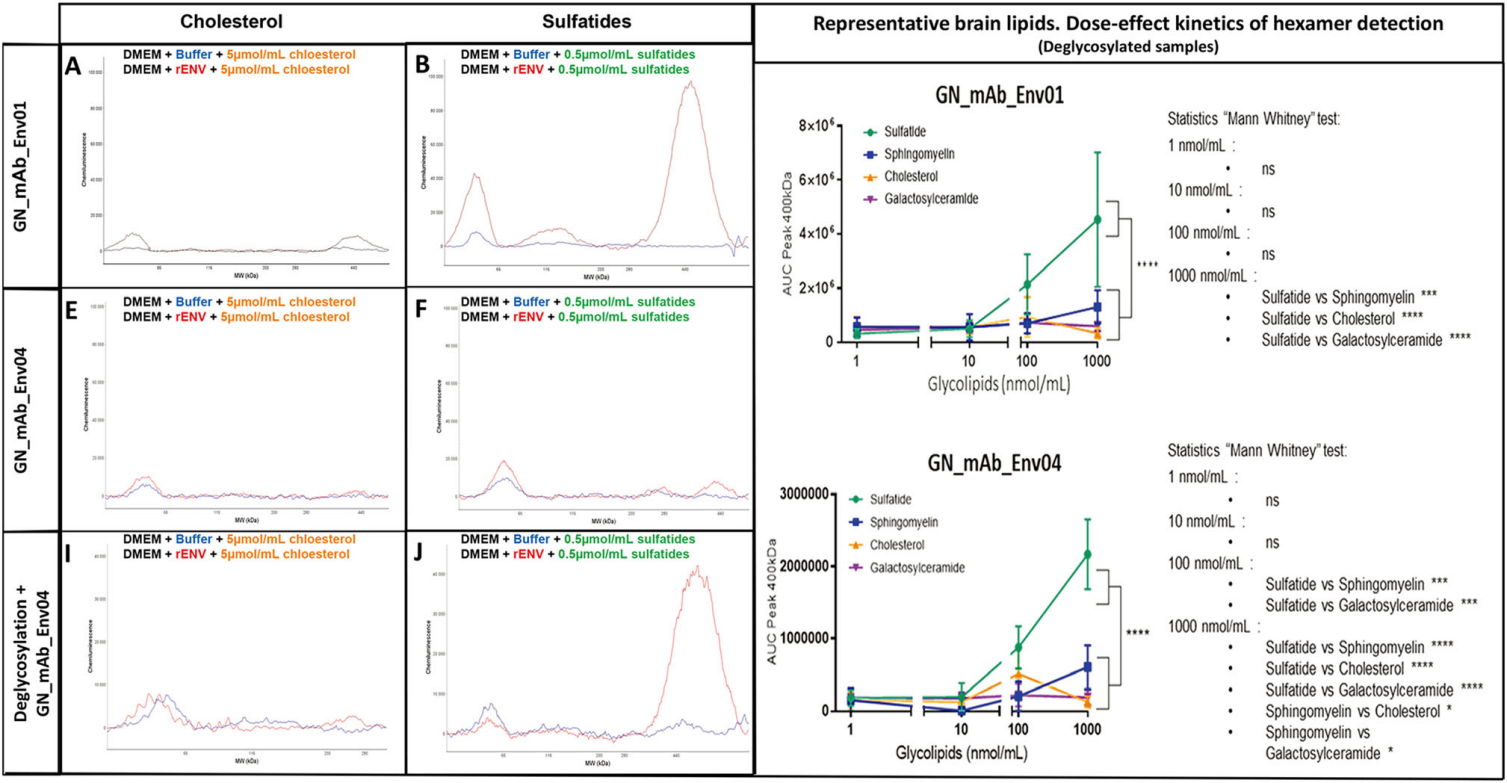
Sulfatides can be associated during pHERV-W ENV hexamer structure formation. Self-assembly properties of purified pHERV-W ENV from *E. coli* expression system (rENV) were assessed in basic DMEM complemented with cholesterol (**A, C, E**) and sulfatides (**B, D, F**). pHERV-W ENV detection was performed with GN_mAb_Env01 (**A, B, G**) or GN_mAb_Env04 (**C–F, H**) antibodies on automated capillary western blot technology (Simple Western®). After incubation with lipids, samples were further deglycosylated (**E, F, H**), or not (**A–D, G**). Monitoring of pHERV-W ENV hexamer formation in presence of increasing concentrations of tested lipids (sulfatide: green, sphingomyelin: blue, cholesterol: orange and galactosylceramide: purple) by WES using GN_mAb_ENv01 (G) or GN_mAb_Env04 (H). Statistical analysis: Tukey’s multiple comparison test (*P < 0.01, **P < 0.05, ***P < 0,001; ****P < 0.0001). Experiment was performed 3 times. Each experiment was repeated 6 times. Profiles are presented as migration electrophoregrams (**A, D** and **G**) or digital western blots (**B, E** and **H**). Quantitative study based on AUC calculation in the area of interest (black square, 200–500 kDa) was performed on non-deglycosylated (**A–F**) or glycosylated brain protein extracts (**G–I**). Statistical analysis: Mann Whitney test (*P < 0.01, **P < 0.05, ***P < 0,001; ****P < 0.0001). n.s. represents non-significant difference. All experiments were repeated 3 times.

To further study other major brain lipids versus sulfatides and their association with pHERV-W ENV hexamer structure, we incubated recombinant pHERV-W ENV monomers in serum-free DMEM complemented with a physiological concentration of cholesterol (Fig. 8A, 8C, 8E), sulfatides (Fig. 8B, 8D, 8F) sphingomyelin (Supplementary Figure S4A, S4C, S4E) and galactosylceramide (Supplementary Figure S4B, S4D, S4F). Hexamer formation was monitored using WES device. Detection with anti-SU GN-mAb_Env01 showed that cholesterol (Fig. 8A) like other tested lipids did not initiate hexamer formation, while sulfatides induced a huge quantity of hexamer (Fig. 8B). Sphingomyelin seemed to initiate low pHERV-W ENV hexamer formation but kinetics with parallel dilution series evidenced a significant yield of hexamers at physiological concentrations with sulfatides only (Fig. 8G–8H). Therefore, cerebrosulfatides can be involved in the thermodynamics of hexamer formation from monomers, even in protein (FCS)-free medium previously shown not to allow such oligomerization. They did not simply interact with preformed soluble hexamers but appeared to confer watertight-like properties to pHERV-W ENV hexamers, in which the SO_4_^2−^ residues should also generate strong electrostatic interactions. Thus, the negative charges of small sulfatide molecules bound to the hexameric structure should neutralize positive charges of SU units and contribute to a lower pHi of brain hexamers, more stable at physiological pH. Sulfatides could be another factor facilitating the solubility of this hexameric pHERV-W antigen by modifying its peptide electropositive surface charge, which would avoid interactions with negatively charged molecules of the extracellular environment.

We further addressed the effects of sulfatides on the biochemical signature of pHERV-W hexamers from MS brain lesions. Since this was previously revealed by the impossibility for the anti-TM GN_mAb_Env04 antibody to reach its target epitope without a prior deglycosylation step (Fig. 6D–6I), we used this *in vitro* model of sulfatide-dependent oligomerization to analyze immunodetection with GN_mAb_Env04 before and after the deglycosylation step (Fig. 8D, and 8E–8F). Results showed that TM epitope detection of pHERV-W ENV hexamer formed in presence of sulfatides was not possible without deglycosylation, mimicking the phenomenon observed with pHERV-W hexamers from MS brain lesions.

Finally, to characterize the relative affinity of sulfatides versus other brain lipids, pHERV-W ENV hexamer formation was monitored on WES, after deglycosylation and using GN_mAb_Env01 (Fig. 8G) or GN_mAb_Env04 (Fig. 8H). This showed that the amount of hexameric pHERV-W ENV forms increased with sulfatides concentration above 10 nmol/mL whereas this oligomer was weakly detectable in presence of sphingomyelin at 1000 nmol/mL and not with the others glycolipids even at high concentrations. The results thus confirmed that, at equal concentration, only sulfatides were very significantly incorporated during the pHERV-W ENV hexamer formation compared to other major brain lipids.

These observations indicate that sulfatides may “close” the external hexameric structure interacting with SU moieties and thereby explain an impossible antibody detection of TM epitopes in the core of native antigen extracted from MS (Fig. 5D). Such sulfatides may originate from degraded myelin sheaths of demyelinating lesions in which they were shown to be decreased, while showing an elevated accumulation in tissue lesions compatible with their molecular dissociation from the myelin structures followed by their release in the extracellular space (Malone and Conole 1969; Wilson and Tocher 1991; Moyano *et al.* 2013; Suchanski and Ugorski 2016).

## Discussion

The present findings reveal the complexity of pHERV-W ENV antigens, suggesting that our previous view of a simple monomeric protein, mostly exposed at the surface of cell membranes and of circulating extracellular vesicles or virus-like particles, was limited. Present results demonstrate that this HERV-W protein in MS brain lesions is not Syncytin-1, but corresponds to the expected protein encoded by sequences obtained from MS-associated retroviral particles, as suggested from our previous work (Perron *et al.* 1997b, 2001; Komurian-Pradel *et al.* 1999). Importantly despite its complexity, its macromolecular soluble form is readily detected by anti-SU monoclonal antibodies known to neutralize its pathogenic effects (Curtin *et al.* 2015).

### Physico-chemical Properties

The present study shed light on unexpected biochemical and physical properties that should be taken into account for future research studies and development projects in the domain.

An unusual oligomeric pattern was observed, as evidenced by capillary immunoelectrophoresis (WES platform) analyses of both recombinant or tissue-extracted proteins, which revealed the existence of monomeric, trimeric and hexameric forms. Though trimeric structures could be predicted by a functional site of trimerization as in exogenous retroviral envelope proteins, it was found that the formation of hexameric structure occurred directly from monomers, even when non-glycosylated and without cleavage of the signal peptide in *E. coli* recombinant full-length protein. This was confirmed by the kinetics of hexamer formation paralleling the decrease of monomers in an appropriate medium with 10% FBS. The detection of oligomers with molecular weights (MW) consistent with a cleavage of the signal peptide in a human cell line transfected with the reference “MSRV” clone encoding pHERV-W ENV, confirmed the production of matured proteins with membrane-associated monomers and trimers along with an important presence of soluble hexamers. The possible production of non-glycosylated forms was also shown and may reflect experimental conditions in transfected cells, but requires further exploration, as the relative glycosylation profiles may provide molecular signatures known to be relevant for other pathogenic “endogenous” proteins such as prions (Ironside and Head 2004; Xanthopoulos *et al.* 2009). In this regard, the ≈120 kDa peak from transfected cells consisted of a double peak containing both the glycosylated monomer and an oligomer with MW of a non-glycosylated dimer. After deglycosylation, a shift toward the peak of deglycosylated monomer and a stable peak at 120 kDa confirmed the presence of both forms. This dimer was shown to result from the partial degradation of the hexamer under denaturing condition, suggesting that non-glycosylated monomers represent a part of the soluble hexamers. However, in native conditions and after deglycosylation, the 120 kDa peak was not observed (Supplementary Figure S3) showing the dimer itself is not directly produced by cells and that it must not be considered as a natural oligomer.

It further appeared that these oligomers have diverging solubility. The monomers and trimers were found in the insoluble phase whereas the hexamers were in the soluble phase. The solubility of the hexameric form was understood to require a specific structure, in which only hydrophilic moieties were exposed to the ambient aqueous medium, thereby burying hydrophobic moieties in a non-exposed core. This can prevent a major instability in solution due to repulsive interactions between hydrophobic regions of pHERV-W ENV and the surrounding hydrophilic medium. The observation that only hexamers were detected in the soluble fraction extracted from MS brain lesions with main characteristics of recombinant expression of the original “MSRV” clone, confirmed their existence as “natural” pHERV-W ENV antigens expressed in human individuals with MS.

As shown with specific monoclonal antibodies targeting distant epitopes on the SU and TM moieties, this structure is maintained despite denaturation treatment, and remains after a further heat-denaturation step in reducing conditions followed by deglycosylation. The observed MW of oligomers also confirmed that the SU and TM units are still contiguous in pHERV-W ENV, whereas they are normally separated by furin proteolytic cleavage in retroviral glycoproteins including syncytin-1, its physiologically co-opted sister protein from HERV-W family. The uncleaved pHERV-W ENV protein represents a hydrophilic-hydrophobic dipole that does not remain solubilized in aqueous media, when present as monomers or trimers. These can be stabilized in cells, virus-like particles or vesicles when anchored into lipid bilayers via their TM membrane spanning domain, which is consistent with immunostaining results from expressing cells and with their presence in proteins from pelleted insoluble particles and cells.

In addition to the absence of furin cleavage, specific monoclonal antibodies targeting distant epitopes on the SU and TM moieties revealed a highly stable hexameric structure formed by uncleaved monomers. From a biophysical standpoint, only hydrophobic forces generate such potent repulsive interaction in aqueous media along with bonds between molecules. This would explain observations of spontaneous hexamer formation from recombinant monomers and the stability of hexamers after thorough denaturation and deglycosylation.

pHERV-W ENV contains only a few glycosylation sites that contribute to a minor molecular load on pHERV-W monomers. However, a significant MW shift was seen at 58 kDa after deglycosylation of the 120 kDa monomer, while hexameric forms persisted after deglycosylation, but with a significant MW shift consistent with loss of glycans. It is therefore possible that hexameric glycoforms may appear superimposed in the enlarged peak of hexamers observed with present WES matrices and capillaries.

The biophysical properties leading to the observed formation of hexamers from pHERV-W ENV monomers were revealed using another envelope protein from HERV-K family, which displayed a better hydrophilic index, as shown by analyses of its amino-acid sequence along with numerous glycosylation sites. The MW shift after deglycosylation of HERV-K ENV monomer from transfected cells confirmed an important glycosylation load. After extraction in denaturing and reducing conditions the observed MW also indicated an effective cleavage of SU and TM units. Oligomeric structures were not observed with HERV-K ENV (cleaved SU), which contains no hydrophobic C-term extremity similar to pHERV-W ENV. To reproduce the “hydrophilic-hydrophobic” dipole properties of pHERV-W ENV, a highly hydrophobic polypeptide (HP) was expressed in fusion at the C-term end of HERV-K ENV. Experiments with this construct confirmed the occurrence of strong hydrophobic interactions between “HERV-K + HP” fusion monomers in aqueous media, since they acquired properties of hexameric oligomerization. The structure–function underlying the highly stable hexameric oligomerization of pHERV-W ENV was thus experimentally evidenced to be mediated by such a hydrophobic extremity. Hexamers certainly represent the most stable forms in aqueous media since burying the hydrophobic TM (C-term) regions while forming a core covered by a hydrophilic surface with SU units in all directions (3D).

Another important and unusual biophysical property of pHERV-W ENV peptide sequence is its electropositivity at physiological (neutral) pH with an isoelectric pH (pHi) above 9. This implicitly generates electrostatic interactions with other naturally electronegative proteins or molecules when expressed *in vivo* and, along with potential hydrophobic interactions, should explain the observed masking effect of albumin with elevated concentrations as observed in serum. This is consistent with the formation of multimolecular complexes in tissues or body fluids, as shown to be partially dissociated with fos-cholin 16, a detergent with a polar extremity and long aliphatic (hydrophobic) chain allowing disruption of both electrostatic and hydrophobic bonds. This may explain many of the difficulties when examining the presence of pHERV-W ENV in blood, where albumin represents 50% of total serum proteins. Other peculiar effects resulting from pHERV-W ENV biophysical properties were described in supplementary material, such as a potent binding to electronegative plastic or glass surfaces, which should be taken into account as a cause of depletion from test samples when using immunoassays to detect pHERV-W ENV as a biomarker in body fluids. Globally, data from presented analyses now provide a clear explanation for the difficulties encountered in developing a routine test for the detection of pHERV-W ENV antigen in body fluid samples, but unexpected features identified within “natural” pHERV-W hexamers from MS brains and detailed from reference clone expression, may offer new perspectives for its detection in a soluble phase in blood.

### Soluble pHERV-W ENV Hexamer with Molecular Specificities is Expressed in Demyelinating MS Brain Lesions

Protein analysis using the WES platform allowed straightforward detection of the pHERV-W ENV hexamer in the soluble phase extracted from frozen tissue blocks of actively demyelinating MS brain lesions, but not in MS normal appearing white matter and non-MS white matter. Soluble hexamers were readily detected with the “pHERV-W ENV-specific” anti-SU antibody shown not to detect syncytin-1. However, unlike in hexamers from transfected cells, the specific anti-TM monoclonal showed no immunodetection at this first step. Deglycosylation of MS pHERV-W ENV hexamers modified the structure and accessibility of TM epitopes since detected by the same anti-TM antibody in a maintained hexameric form. Immunodetection of pHERV-W ENV hexamer from MS brain lesions with anti-SU and anti-TM specific monoclonal antibodies occurred within in the same MW range when tested in parallel capillaries, with an enlarged peak mostly due to lower resolution of the matrix for high MW.

The original biophysical property precluding the detection by anti-TM antibody in non-deglycosylated soluble hexamer from MS lesions was elucidated: hexamer formation in presence of brain sulfatides reproduced the characteristics of native MS-antigen. The rationale for an interaction with sulfatides and the resulting hermetic tightening of the hexameric structure from MS brains could explain a better solubility of the “natural” MS antigen, only exposing hydrophilic SU and sulfatides molecules at the surface of a closed sphere-like structure, as illustrated in Fig. 5D. This explains the need to deglycosylate this complex structure in order to gain access to the internalized TM epitopes with the anti-TM antibody, since the conditions of deglycosylation (cf. Materials and Methods) allow the cleavage of glycans from both SU glycosylations and sulfatides. Molecular bonds between sulfatides and pHERV-W ENV hexamer are therefore likely to require glycans. These glycolipids may thus represent robust candidates contributing to the specific signature of pHERV-W ENV soluble hexamers from active lesions of MS.

Consequently, demyelination itself may confer a higher solubility to secreted pHERV-W ENV hexamer when releasing sulfatides from degraded myelin sheaths into the extracellular space or in microglia intracellular vesicles within which hexamers may also be formed, as suggested by immunohistology studies of MS brain lesions (Kremer *et al.* 2013; van Horssen *et al.* 2016).

Despite its original characteristics and the most probable association with sulfatides, the pHERV-W ENV hexamer was readily detected by the specific anti-SU monoclonal antibody, GN-mAb-Env01, from which CDR-encoding sequences were used to generate the pHERV-W ENV neutralizing therapeutic antibody, temelimab -formerly GNbAC1- (Curtin *et al.* 2015). Temelimab targets the identical epitope with high affinity in SU moieties of soluble hexameric pHERV-W antigen that likely represents the soluble “neurotoxic” factor incriminated in sub-pial demyelination and produced by B-cells (Lassmann 2014; Lisak *et al.* 2017). Thus, observed positive effects of temelimab on neurodegeneration and on myelin loss in recent clinical trials (ClinicalTrials.gov Identifier: NCT02782858; Manuscript “in revision”, on phase II 1&2-years results) may be due to the neutralization of this neuro- and glio-toxic soluble hexameric form.

The immunolabelling of pHERV-W ENV hexamers in protein extracts of active MS lesions revealed the presence of distant epitopes representing diverging amino acid sequences from syncytin-1, using monoclonal antibodies not detecting syncytin-1 in parallel analyses. Together, with the finding of an absence of furin cleavage and of fusogenic activity, immunolabelling and biochemical data confirm that the pHERV-W ENV antigen detected in MS lesion is not syncytin-1.

All original physico-chemical and immunological characteristics defining pHERV-W ENV antigen and its soluble hexameric form were obtained from examining the “natural” MS antigen. Only additional interaction with brain glycolipids required to deglycosylate the antigen before detection with specific anti-TM monoclonal antibody, which may confer additional properties to MS soluble hexamers. Thus, the elucidation of the molecular structure of the pHERV–W antigen must be important for routine diagnostic applications that failed to address the unique properties of this antigen with usual protocols in, e.g., immunoassays or even mass spectrometry.

Elsewhere, since the corresponding specific nucleotide sequences were not recorded in present human genome databases, this highlights an important need for pursuing the search of non-ubiquitous/unfixed or somatically modified HERV-W copies in pHERV-W ENV expressing cells from active MS lesions (Kremer *et al.* 2019b; Ruprecht and Mayer 2019). This is now known to require newly defined cloning and sequencing approaches, as present sequencing techniques and platforms cannot appropriately deal with numerous and highly homologous multicopy elements spread into chromosomal DNA; especially when potentially representing nearly 10 Kb flanked by two identical long inverted repeated sequences (LTR) (Huddleston *et al.* 2017; Ardui *et al.* 2018; Sedlazeck *et al.* 2018; Thomas *et al.* 2018; Mantere *et al.* 2019). Numerous and highly similar LTRs add to the difficulty and, when flanking coding proviral sequences, may be taken as a single sequence contiguous to both insertion sites in a given chromosomal locus. This often results in eliminating internal proviral sequences further affected to the “trash” as redundant reads homologous to many other HERV copies (Bourque *et al.* 2018; Thomas *et al.* 2018; Zhang *et al.* 2018). Moreover, new cloning and/or sequencing protocols should also be used to detect eventual somatic rearrangements in expressing cells of affected tissue, which would not be present as inherited sequences in other cells (Iourov *et al.* 2010).

Finally, pHERV-W ENV was detected by immunohistochemistry in MS brain post-mortem tissue—86 cases published to date (Antony *et al.* 2004; Perron *et al.* 2005; Perron *et al.* 2012; Kremer *et al.* 2013, 2019a; van Horssen *et al.* 2016; Gottle *et al.* 2019) and has now been confirmed to be present as a dominant soluble high molecular weight hexameric oligomer in MS lesions. Given the pathogenic properties of pHERV-W ENV (Kremer *et al.* 2013; Kremer *et al.* 2019a; Perron *et al.* 2013) and its known expression from MS B-cells (Haahr *et al.* 1991; Perron *et al.* 1997a; Firouzi *et al.* 2003), the question is now raised about a possible identity between this soluble pHERV-W ENV hexamer and the previously described “high-molecular weight” demyelinating/neurotoxic factor in MS brains (Lassmann 2014; Lisak *et al.* 2017). Addressing this question may also elucidate major pathogenic pathways relevant to disease progression in MS providing another rationale for targeting this soluble neurotoxic antigen with a therapeutic antibody.

## Supplementary Information

Below is the link to the electronic supplementary material.

Supplementary file1 (PDF 412 kb)

## Abbreviations

AA: Amino Acid
CHCl_3_: Chloroform
CHR: C-Heptad Repeats
CMV: CytoMegaloVirus
CYT: Intracytoplasmic tail
ELISA: Enzyme-Linked ImmunoSorbent Assay
ENV: Envelope
FBS: Fetal Bovine Serum
GM1: Monosialoganglioside
GB1a: Bisialoganglioside
GT1b: Trisialoganglioside
HERV: Human Endogenous RetroVirus
HP: Hydrophobic peptide
IM: Immunosuppressive domain
MetOH: Methanol
MS: Multiple Sclerosis
MSRV: Multiple Sclerosis RetroVirus
MW: Molecular Weight
NAWM: Normal Appearing White Matter
NHR: N-Heptad Repeats
ON: Over Night
ORF: Open Reading Frame
T1D: Type 1 Diabetes
TM: TransMembrane Unit
SP: Signal peptide
SU: Surface Unit
WM: White Matter
