# Supplementary material for: Human Endogenous Retrovirus Type W Envelope from Multiple Sclerosis Demyelinating Lesions Shows Unique Solubility and Antigenic Characteristics
Source: Virol Sin. 2021 Mar 26;36(5):1006–26. doi: 10.1007/s12250-021-00372-0 (PMC8558138; doi:10.1007/s12250-021-00372-0)
Supplement: Supplementary file 1 — Supplementary file1 (PDF 412 kb) [file 12250_2021_372_MOESM1_ESM.pdf]

VIROLOGICA SINICA

Electronic Supplementary Material

Human Endogenous Retrovirus Type W Envelope from Multiple Sclerosis Demyelinating Lesions Shows Unique Solubility and Antigenic Characteristics

Benjamin Charvet[1,2,3]✉ • Justine Pierquin[1] • Joanna Brunel[1,2,3] • Rianne Gorter[4] • Christophe Quétard[5] • Branka Horvat[2,3] • Sandra Amor[4,6] • Jacques Portoukalian[3] • Hervé Perron[1,3]✉

1. GeNeuro Innovation, Lyon 69008, France
2. CIRI, International Center for Infectiology Research, INSERM U1111, CNRS UMR5308, University of Lyon, ENS Lyon, France
3. Université Claude Bernard Lyon 1, Lyon 69000, France
4. Department of Pathology, Amsterdam UMC, location VUMC 1007 MB Amsterdam, The Netherlands
5. ProteinSimple, Abingdon OX14 3NB, UK
6. Centre for Neuroscience and Trauma, Blizard Institute, Barts and London School of Medicine and Dentistry, Queen Mary University of London, London E1 2AT, UK

Supporting information to DOI: 10.1007/s12250-021-00372-0

Sequence of « **Hydrophobic peptide (HP)** »:

MKRQKIHFYFNCSDYGINCSHSYGCCSRSCIALFCSVSKLC

Sequence of HERV-K ENV-**HP**:

MNPSEMQRKAPPRRRRHRNRAPLTHKMNKMVTSEEQMKLPSTKKAEPPTWAQLKKLTQLATKYLENTKVTQTPESMLLAALMIV
SMVVSLPMPAGAAAANYTYWAYVPFPPLIRAVTWMDNPIEIYVNDSVWVPGPTDDCCPAKPEEEGMMINISIGYRYPPICLGRA
PGCLMPAVQNWLVEVPTVSPISRFTYHMVSGMSLRPRVNYLQDFSYQRSLKFRPKGKPCPKEIPKESKNTEVLVWEECVANSAV
ILQNNEFGTLIDWAPRGQFYHNCSGQTQSCPSAQVSPAVDSDLTESLDKHKHKKLQSFYPWEWGEKGISTARPKIISPVSGPEH
PELWRLTVASHHIRIWSGNQTLETRDRKPFYTIDLNSSLTVPLQSCVKPPYMLVVGNIVIKPDSQTITCENCRLLTCIDSTFNW
QHRILLVRAREGVWIPVSMDRPWEASPSVHILTEVLKGVLNRSKR**QKIHFYFNCSDYGINCSHSYGCCSRSCIALFCSVSKLC**

Fig. S1 Amino-acid and nucleotide sequences of the hydrophobic peptide (HP) and of the HERV-K ENV-HP chimeric protein. HP amino acids within the fusion protein (HERV-K ENV SU+HP) are indicated with bold letters.

A

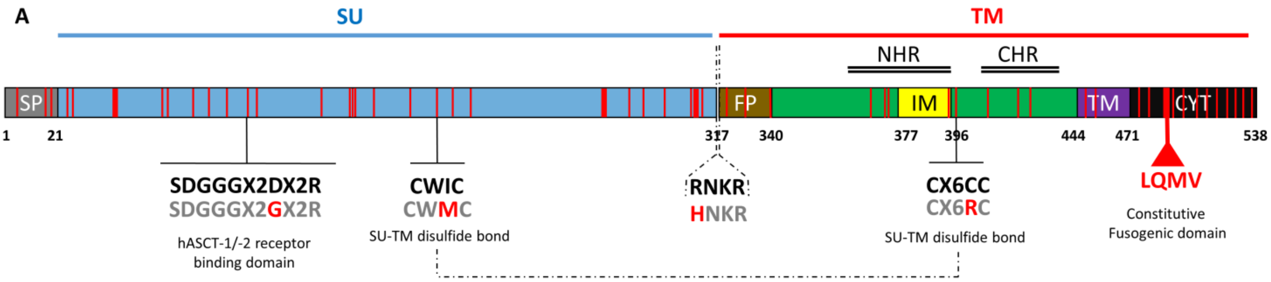

B

Functional domain	syncytin-1	pHERV-W ENV	aa mutated position
hASCT-1/-2 receptor binding domain	SDGGG**V**Q**D**QAR	SDGGG**I**Q**G**QAR	120-122
SU-TM disulfide bond formation	CW**I**C	CW**M**C	188
Furin cleavage site	**R**NKR	**H**NKR	314
Fusion peptide	VPILPFVI**G**AGVLG**A**LGTGIG**G**I	VPILPFVI**R**AGVLG**R**LGTGIG**S**I	326-332-339
N-heptad repeats (NHR) - fusion core	QE**L**NGDME**R**V**A**DSLVTLQDQLNSLAAVVLQNRRALDLLTA**E**	QE**I**NGDME**Q**V**T**DSLVTLQDQLNSLAAVVLQNRRALDLLTA**K**	354-360-362-392
putative immunosuppresive domain	AAVVLQNRRALDLLTA**E**R	AAVVLQNRRALDLLTA**K**R	395
SU-TM disulfide bond formation	CX6**C**C	CX6**R**C	405
C-heptad repeats (CHR) - fusion core	VNQS**G**IVTEKVKEIRDRIQ**R**RAEEL**R**NT**GP**WGLLS	VNQS**R**IVTEKVKEIRDRIQ**C**RAEEL**Q**NT**ER**WGLLS	412-427-433-436-437
membrane anchorage domain	MPW**I**LPFLGPLAAII**L**LLLFGPCIFN	MPW**T**LPFLGPLAAII**F**LLLFGPCIFN	448-460
constitutive fusogenic activity domain	EAVK**----**LQMEP	EAVK**LQIV**LQMEP	485-486-487-488

Fig. S2 Schematic comparison of amino-acid sequence between syncytin-1 and pHERV-W ENV**.** **A** Schematic representation of syncytin-1 (black letters sequences) presenting main diverging clusters as aligned nucleotide sequences with pHERV-W ENV (grey letters sequences). Other "mutations" on pHERV-W ENV sequence compared to syncytin-1 sequence are represented by red dashes or by red letters in the corresponding region. SU: Surface Unit, TM, TransMembrane unit, SP: Signal Peptide, FP: Fusion Peptide, IM: IMmunosuppressive domain, NHR: N-Heptad Repeats, CHR: C- Heptad Repeats, CYT: intraCYToplasmic domain. **B** Recapitulative table of main functional domains of HERV-W family envelopes. Amino-acid sequences of each domain are mentioned for syncytin-1 and pHERV-W ENV, and differences are highlighted in bold with indicated position of mutations.

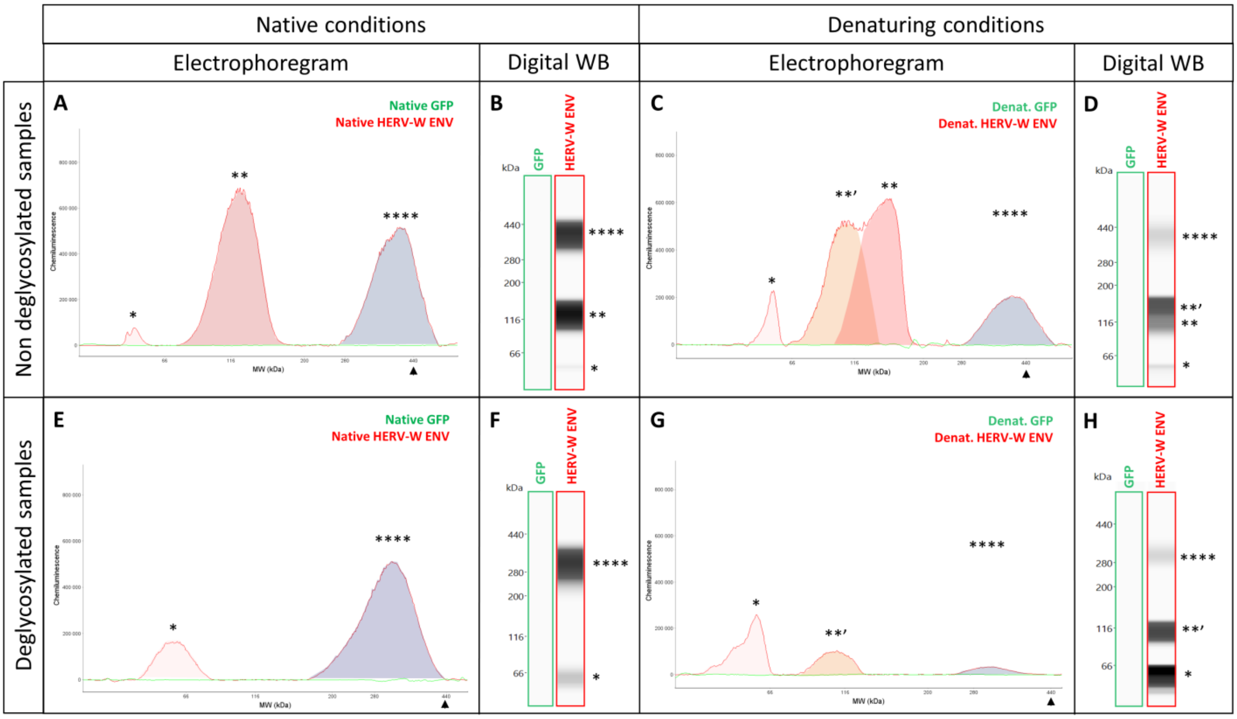

Fig. S3 Deciphering pHERV-W ENV 120 kDa signal composition. Lysates of HEK293T cells transfected with sequences encoding GFP (green panels) or pHERV-W ENV (red panels) were analyzed by Simple Western®, using GN_mAb_Env01 antibody, on 66-440 kDa size separation matrix. Native (**A, B**) and denatured (**C, D**) soluble fractions from transfected cells lysates are compared on electrophoregrams (**A, C, E, G**) or digital western blot (**B, D, F, H**) representations. These native (**E, F**) and denatured (**G, H**) protein extracts are also compared after deglycosylation in order to separate the glycosylated pHERV-W ENV monomer, sensible to deglycosylation (red AUC), from the dimer (orange AUC) which is generated by the degradation of the hexamer under denaturing conditions, whereas not appearing to be sensible to deglycosylation under the present limits of detection. *: pHERV-W ENV monomer, **: glycosylated pHERV-W ENV monomer, **': pHERV-W ENV dimer, ****: pHERV-W ENV hexamer. Because position of size marker are depending upon the protein migration in each capillary, black arrow head highlight the 440 kDa size marker in order to appreciate the mass shift of the hexamer after deglycosylation. Each experiment was repeated 3 times.

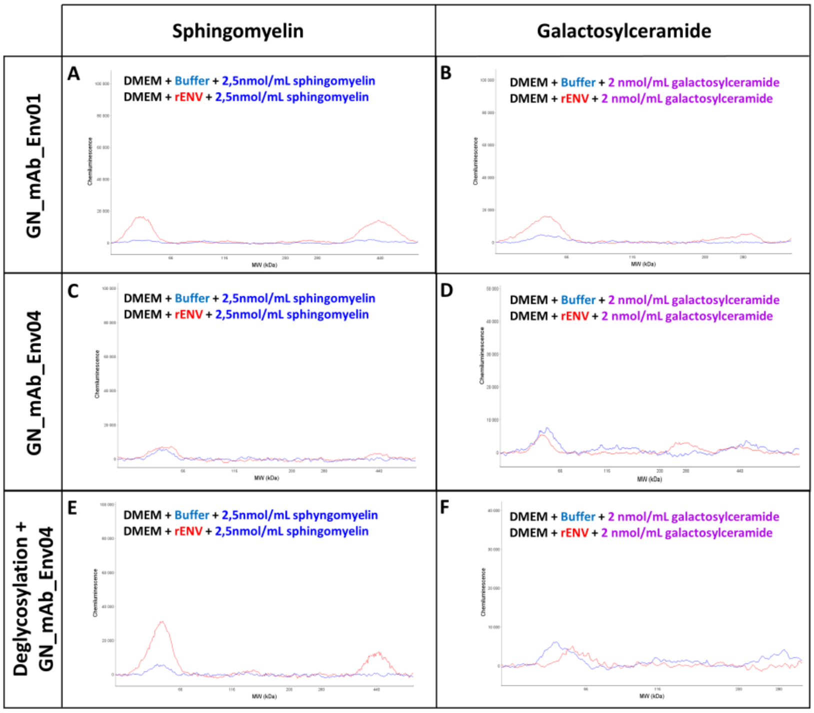

Fig. S4 Absence of involvement of brain lipids other than sulfatides, in pHERV-W ENV hexamer formation. The self-assembly properties of purified pHERV-W ENV from E. coli expression system (rENV) were assessed in basic DMEM complemented with sphingomyelin (**A, C, E**) and galactosylceramide (**B, D, F**). pHERV-W ENV detection was performed with GN_mAb_Env01 (**A, B**) or GN_mAb_Env04 (**C–F**) antibodies on automated capillary western blot technology (Simple Western®). After incubation with lipids, samples were deglycosylated (**E, F**), or not (**A–D**). Each experiment was repeated 6 times.
